# Hypertrophic Cardiomyopathy: Current Perspectives

**DOI:** 10.31083/RCM42824

**Published:** 2025-09-12

**Authors:** Dhruvil Patel, Ruchika Bhargav, Aliaa Mousa, Sabahat Bokhari

**Affiliations:** ^1^Department of Cardiology, Rutgers Robert Wood Johnson University Hospital, New Brunswick, NJ 08901, USA

**Keywords:** hypertrophic cardiomyopathy, sudden cardiac death, alcohol septal ablation, surgical myectomy, mavacamten, aficamten

## Abstract

Hypertrophic cardiomyopathy (HCM) is a multifaceted genetic disorder characterized by left ventricular hypertrophy (LVH) in the absence of alternative causes, with an estimated prevalence ranging from 1 in 200 to 1 in 500 individuals. Since HCM was first characterized in 1869, a plethora of pathogenic mutations have been identified, while ongoing research continues to elucidate the various pathophysiological mechanisms present in individuals with HCM. Comprehensive physical examination findings and multimodality imaging techniques have become crucial for accurately diagnosing and risk stratifying HCM patients. Meanwhile, recent advancements in research have contributed to a more refined definition and heightened recognition of HCM, prompting further investigations into targeted therapeutic strategies. This evolution in understanding provides alternative treatment options for patients, moving beyond traditional approaches such as myectomy or septal ablation. This review aims to systematically explore the genetic and pathophysiological underpinnings of HCM, as well as the application of multimodality imaging in identifying patients at risk for sudden cardiac death (SCD). The discussion also examines contemporary management strategies for HCM, specifically highlighting novel therapies targeting the molecular mechanisms involved in this disease.

## 1. Introduction

The understanding of hypertrophic cardiomyopathy (HCM) as a distinct clinical entity has developed over a century, beginning with early morphological descriptions and culminating in recognizing its genetic basis. HCM is a genetic disease of the myocardium, which is characterized primarily by left ventricular hypertrophy (LVH) that is not due to alternative systemic, cardiac, or metabolic etiologies.

The earliest descriptions of HCM date back to Henri Liouville in 1869, who characterized the disease as “cardiac subaortic stenosis” after he discovered massive concentric LVH to 3.5 cm and left ventricular outflow tract obstruction in an autopsy study of a 75-year-old female [1]. Throughout the late 19th and early 20th centuries, case reports and pathological studies documented unexplained myocardial hypertrophy, but these observations remained isolated and lacked understanding of the underlying disease mechanisms [2, 3]. In 1958, Teare and colleagues provided a comprehensive description of familial cases characterized by massive septal hypertrophy, myocyte disarray, and sudden cardiac death, marking the first recognition of HCM as a hereditary disease [4]. Teare’s studies emphasized that the condition was characterized by familial clustering and involved disorganized myocardial architecture with myocyte disarray [4].

HCM is now recognized as a relatively common inherited cardiac disorder with a worldwide distribution. Recent epidemiological studies, supported by advanced echocardiography and cardiac magnetic resonance imaging (CMR), suggest a population prevalence of approximately 1 in 200 to 1 in 500 individuals [5]. From a morphological perspective, HCM is characterized by the restriction of hypertrophy to the myocardium. While it often exhibits asymmetric septal hypertrophy, it can also present with concentric or localized hypertrophy, including apical involvement. The disease may progress from compensated hypertrophy to restrictive cardiomyopathy and end-stage heart failure due to myocardial remodeling and fibrosis. The clinical diagnosis considers genetic and phenotypic features, emphasizing the importance of integrating imaging, electrocardiographic, genetic, and clinical data for an accurate diagnosis [6, 7, 8].

From a genetic standpoint, HCM was initially solely considered to be an autosomal dominant disorder and determined to be caused by pathogenic variants in sarcomere protein genes, including *MYH7* (β-myosin heavy chain) and *MYBPC3* (cardiac myosin-binding protein C), which are responsible for roughly 60–70% of familial cases [9]. Mutations in these genes tend to lead to more severe disease phenotypes and manifest earlier in life, especially with specific variants like p.Arg403Gln in *MYH7* [9]. Other less common but implicated genes include *TNNT2* (Troponin T), *TNNI3* (Troponin I), *TPM1* (α-tropomyosin), *ACTC1* (α-cardiac actin), *MYL2* (myosin light chain 2), *MYL3* (myosin light chain 3), and *CSRP3* [9].

However, there is increasing data regarding autosomal recessive inheritance, especially in populations where consanguinity is more prevalent. Pathogenic variants are responsible for approximately 30%–40% cases of HCM. Less prevalent causal genes such as *MYL2*, *MYL3*, *CSRP3*, and *TRIM63* have been implicated in more homozygous cases [10]. Table 1 (Ref. [10]) highlights the common genetic mutations and their prevalence.

**Table 1.** **Common genetic variants in hypertrophic cardiomyopathy [10]**.

Gene	Protein encoded	Frequency within genotype—positive individuals
*MYBPC3*	Myosin-binding protein C	40%–50%
*MYH7*	Beta-myosin heavy chain	35%–40%
*TNNT2*	Troponin T	7%–15%
*TNNI3*	Troponin I	5%
*TPM1*	Tropomyosin	3%
*MYL2*	Regulatory myosin light chain	1%–2%
*MYL3*	Essential myosin light chain	1%
*ACTC1*	Actin	1%
*TNNC1*	Troponin C	<1%
*ACTN2*	Alpha-actinin-2	<1%
*ALPK3*	Alpha-protein kinase 3	~2%
*FHOD3*	Formin homology 2 domain containing 3	1%–2%
*CSRP3*	Muscle LIM protein	<1%
*TRIM63*	Tripartite motif containing 63	Unknown
*FLNC*	Filamin C	<1%
*FHL1*	Four-and-a-half LIM domain protein 1	<1%
*PLN*	Phospholamban	<1%
*JPH2*	Junctophilin 2	Unknown

Additionally, approximately 40% of patients have nonfamilial forms of HCM, which is a clinically distinct subtype and includes probands who have no identifiable genetic cause or family history of HCM. Male gender, older age, lack of asymmetric hypertrophy, and hypertension are more frequently associated with nonfamilial HCM. The nonfamilial HCM subgroup generally has a more benign clinical course with a lower rate of adverse cardiac events as compared to patients with sarcomere-positive HCM [11].

Phenotypic expression can vary significantly: a substantial number of mutation carriers may remain asymptomatic due to incomplete penetrance. Furthermore, even individuals with pathogenic mutations can show considerable variability in disease severity, age of onset, and clinical outcomes, often within the same family. The genetics of HCM involve a combination of strong-effect mutations, polygenic modifiers, and non-genetic factors, highlighting the necessity for comprehensive and nuanced genetic evaluation [5].

This paper aims to provide a comprehensive review of the pathophysiology, screening criteria, diagnosis, and advancements in the treatment of HCM.

## 2. Pathophysiology

### 2.1 Dynamic Left Ventricular Outflow Tract Obstruction

**Fig. 1.**
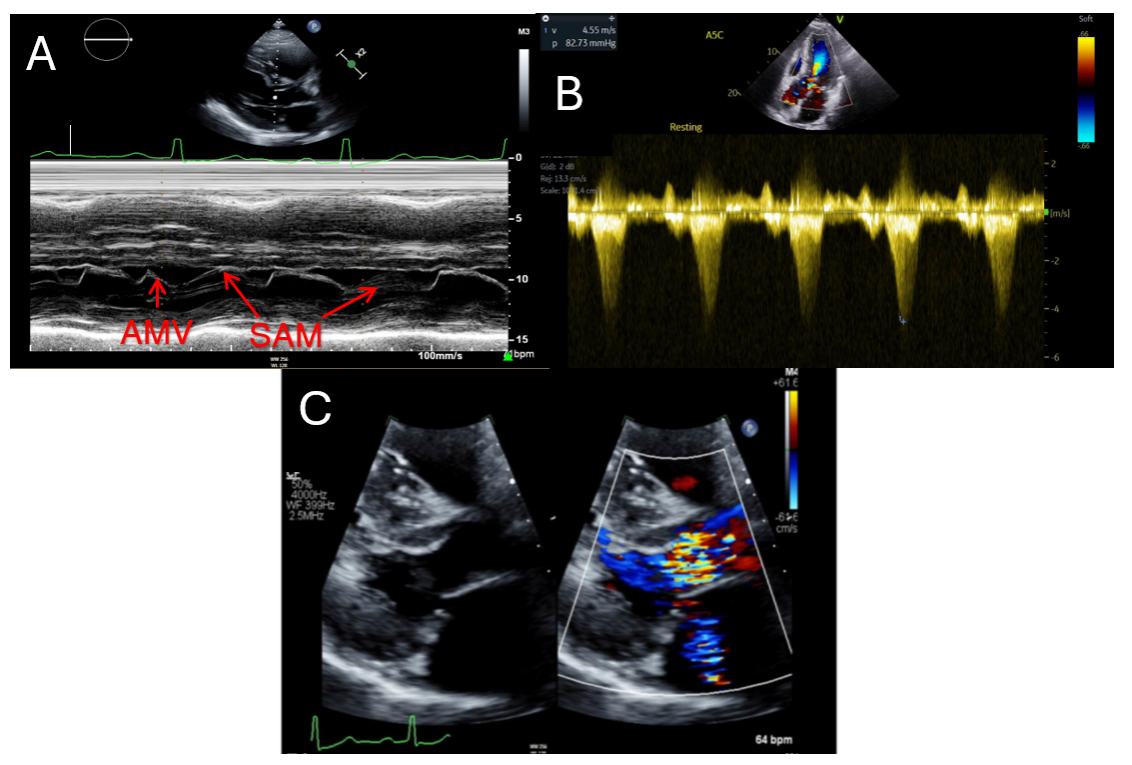
**Echocardiographic findings in hypertrophic cardiomyopathy**. (A) showcases a patient with systolic anterior motion of the anterior mitral valve leaflet (AMV) demonstrated by M-Mode of the parasternal long axis. (B) highlights a severely obstructed outflow tract gradient (82.7 mmHg) in an HCM patient. (C) displays mild mitral regurgitation via color seen on parasternal long axis. HCM, hypertrophic cardiomyopathy; SAM, systolic anterior motion; AMV, anterior mitral valve leaflet.

Left ventricular outflow tract obstruction (LVOTO) represents a hallmark pathophysiologic mechanism in HCM, affecting approximately 70% of patients with gradients ≥30 mmHg at rest or with physical provocation [12]. The obstruction predominantly involves the systolic anterior motion (SAM) of the mitral valve (MV) leaflet, which contacts the hypertrophied interventricular septum (IVS) during systole, creating mechanical resistance to left ventricular (LV) ejection [8] (Fig. 1, Video 1). Ventricular loading conditions influence the dynamic nature of this obstruction. Increased contractility or decreased preload and afterload can exacerbate obstruction by reducing ventricular volume, making HCM with LVOTO a preload-dependent, afterload-sensitive condition [6]. This explains the characteristic variability in gradient severity with postural changes, exercise, pharmacologic agents, and physiologic maneuvers such as Valsalva or squatting, representing a mechanistic foundation for symptomatology and therapeutic interventions in obstructive HCM (oHCM) [6].

**Video. 1.** **Apical hypertrophic cardiomyopathy visualized by cardiac magnetic resonance imaging**. Video associated with this article can be found, in the online version, at https://doi.org/10.31083/RCM42824.

### 2.2 Mitral Regurgitation

Mitral regurgitation (MR) in patients with HCM primarily occurs due to SAM of the MV, which is commonly attributed to the Venturi effect [13]. However, it can also result from intrinsic abnormalities of the mitral valve apparatus, such as excessive elongation of the anterior or posterior leaflets, and issues with the papillary muscles, including anomalous insertion or anterior displacement [2, 14]. Understanding these pathophysiological changes is crucial, as they can precede hypertrophic changes in the LV and may represent an early phenotypic manifestation of sarcomere mutations [15, 16]. For example, Velicki *et al*. [17] investigated patients with mutations in the *MYBPC3* or *MYH7* genes and found that those with *MYH7* mutations exhibited more significant MV abnormalities and greater regurgitation compared to patients with *MYBPC3* mutations. This genetic heterogeneity may account for the variability in valvular dysfunction observed across the HCM spectrum.

The etiology of MR also carries management implications, as study has demonstrated a significant reduction in MR after isolated septal myectomy without requiring additional MV surgery [18]. In contrast, those with intrinsic disease of the MV apparatus may require an additional MV repair procedure during septal myectomy [2, 18, 19]. Differentiation of the etiology of MR is therefore crucial for pre-procedural planning in patients undergoing septal reduction surgery. While SAM-mediated MR often presents with a posteriorly directed jet, its absence does not definitively rule in primary mitral valve disease due to its low negative predictive value [20, 21]. Conversely, central or anteriorly directed jets should prompt further investigation via transesophageal echocardiogram or cardiac magnetic resonance imaging to evaluate for structural abnormalities [2, 20].

### 2.3 Diastolic Dysfunction

As the disease progresses, many cellular and morphological changes drive diastolic dysfunction in HCM. This occurs due to impaired LV relaxation, increased myocardial stiffness, and left atrial myopathy [8, 20, 22]. Using induced pluripotent stem cell-derived cardiomyocytes (iPSC-CMs) from healthy controls and HCM patients with diastolic dysfunction, Wu *et al*. [22] showed that diastolic calcium overload, slow calcium recycling, and increased myofilament calcium sensitivity collectively impaired diastolic relaxation times; thereby providing a molecular explanation for the utility of calcium channel blockers (CCBs) in the management of HCM. Myocardial stiffness in HCM, at the cellular level, results from hypertrophied and disorganized cardiomyocytes separated by interstitial fibrosis, leading to a shrinking LV cavity [6]. In severe cases, this may result in a restrictive physiology [2]. Lastly, left atrial myopathy contributes to the development of diastolic dysfunction by impairing LV filling [20]. A culmination of these three pathophysiologic mechanisms results in elevated LV diastolic pressures that increase markedly on exertion, resulting in symptoms of exertional dyspnea and exercise intolerance, especially in patients with concomitant atrial fibrillation (AF) [6].

### 2.4 Myocardial Ischemia

The pathophysiology of myocardial ischemia in HCM is complex and involves multiple factors. One mechanism is coronary microvascular dysfunction (CMD), as evidenced by autopsy studies showing hypertrophy of intimal and/or medial layers of the coronary arterioles with resultant reduction in luminal cross-sectional area [23]. The consequent impairment of vasodilatory capacity leads to decreased myocardial blood flow (MBF) during periods of heightened physiological demand, such as exercise, severe hypertrophy, and significant LVOTO. Consequently, this creates a pronounced myocardial supply-demand mismatch and myocardial injury [23, 24, 25]. Therefore, if this mismatch persists, replacement fibrosis occurs and serves as an arrhythmogenic substrate associated with sudden cardiac death [24, 26].

### 2.5 Arrhythmias

With an estimated prevalence of 20%, AF is the most common sustained arrhythmia in patients with HCM [8]. The elevated LV filling pressures from progressive hypertrophy leading to left atrial dilation have been attributed to the development of AF. However, increasing evidence of a primary left atrial (LA) myopathy secondary to excessive fibrosis may also serve as a predisposing factor for AF [8, 27]. In contrast to atrial arrhythmias, the mechanism behind ventricular arrhythmias in HCM patients is multifaceted and involves both functional and structural abnormalities. Functionally, alterations in intracellular calcium and sodium homeostasis lead to prolonged action potentials in the diseased myocardium, increasing the susceptibility to early and delayed afterdepolarizations that can trigger ventricular arrhythmias [28, 29, 30]. Structurally, myocardial fibrosis, myocyte disarray, and CMD act as substrates for re-entry ventricular tachycardia, with precipitating factors including exercise and LVOTO [31]. Refer to section 6 for additional discussion on managing and preventing arrhythmic complications in HCM.

### 2.6 Metabolic and Energetic Abnormalities

The mitochondrion has been of particular focus at the cellular level for HCM. Recent studies have demonstrated severe mitochondrial damage with additional downregulation of genes responsible for synthesizing creatinine kinase and adenosine triphosphate, suggesting a global energetic decompensation in HCM hearts [32, 33]. This energy deficit was further exacerbated, independent of hypertrophy or degree of fibrosis, during exercise in patients with HCM [34]. These findings underscore the importance of understanding the pathology of HCM at a cellular level to develop effective therapies.

## 3. Diagnosis

### 3.1 Clinical Clues

HCM is predominantly diagnosed in an outpatient clinical setting [4, 5, 6]. Initial workup involves thoroughly assessing a patient’s personal history, obtaining a three-generational family history, and a focused physical exam. In obstructive or labile-obstructive disease variants, patients typically manifest with dyspnea, angina, presyncope, or syncope, with the latter being particularly prevalent among young athletes [6, 13, 35]. Individuals exhibiting a milder phenotype demonstrate attenuated symptomatology, with a subset remaining entirely asymptomatic [6, 13]. For these asymptomatic individuals, a meticulous physical exam and a detailed family history become instrumental in determining the necessity for additional diagnostic imaging. Cardinal examination findings include a leftward-displaced precordial impulse, brisk peripheral artery pulsations, and a pronounced fourth heart sound (S4) [6, 13]. Depending on the obstruction’s severity, a harsh mid-systolic murmur may be best heard between the left lower sternal border and the cardiac apex. A blowing high-pitched holosystolic apical murmur suggestive of mitral regurgitation may also be present [6, 13]. Lastly, augmentation of mid-systolic murmur intensity during preload-reducing maneuvers—specifically the Valsalva maneuver or orthostatic positioning—provides further diagnostic differentiation from aortic stenosis, reinforcing the diagnosis of HCM [35, 36].

### 3.2 Diagnostic Modalities 

#### 3.2.1 Electrocardiogram

The electrocardiogram (ECG) maintains its position as an indispensable screening modality for HCM, with contemporary data indicating that merely 5–10% of affected individuals present with normal electrocardiographic patterns [37, 38]. This cost-effective diagnostic instrument demonstrates utility in detecting left atrial enlargement (LAE), LVH, and Wolff-Parkinson-White (WPW) syndrome [37, 39]. These findings warrant further imaging workup if present on ECG, as they have prognostic implications. For example, an increase in left atrial diameter is associated with an increased risk of sudden cardiac death (SCD). Moreover, significant LVH with WPW is closely linked to PRKAG2 syndrome, a rapidly progressive autosomal-dominant glycogen storage disorder that carries a high risk of arrhythmias and SCD [37, 39]. Furthermore, while ECGs alone are not very sensitive for screening HCM or identifying high-risk features for SCD, recent artificial intelligence (AI) breakthroughs show promise in this area [38, 40, 41, 42]. For example, a study done by Ko *et al*. [38] found that AI-based ECGs can effectively screen for HCM, achieving an overall sensitivity of 87%, specificity of 91%, and a negative predictive value of 99%. Another study found similar results using AI in a significantly younger population of children and adolescents [40, 41]. Despite these technological advances in ECG analysis, definitive HCM diagnosis still necessitates supplementary multimodality imaging assessment.

#### 3.2.2 Echocardiography

Transthoracic echocardiography (TTE) constitutes the cornerstone imaging modality in the diagnostic algorithm for HCM, with maximal LV myocardial thickness ≥15 mm establishing diagnostic criteria in the majority of affected individuals [20]. In patients harboring familial predisposition or pathogenic mutations, characteristic dynamic LVOTO, or pathognomonic electrocardiographic patterns, the diagnostic threshold is reduced to a maximal LV thickness ≥13 mm [5, 43]. Due to variation in LV thickness among different stages of development in pediatric patients, an LV wall thickness with a Z-score >2 constitutes a diagnosis in this population [5, 20]. Through direct visualization, TTE aids in characterizing the patterns of HCM, such as basal septal, mid-septal, mid-ventricular, and apical hypertrophy [43]. Using echo contrast is particularly beneficial for characterizing patients with poor acoustic windows or large moderator bands, as it distinguishes heart borders to assess wall thickness and the distribution of hypertrophy [43]. Notably, the septal morphology observed in HCM subtypes is also predictive of genetic disease, with a higher prevalence identified in patients with a reverse curved septum than a sigmoidal one [20, 44].

Furthermore, TTE offers invaluable hemodynamic assessment capabilities, enabling non-invasive differentiation between obstruction phenotypes. Sustained elevation of LVOT gradient (≥30 mmHg) during resting and provocative conditions definitively characterizes oHCM, whereas gradient elevation exclusively during provocative maneuvers indicates labile-obstructive physiology [20, 43]. Consequently, non-obstructive HCM is characterized by LVOT gradients consistently <30 mmHg irrespective of activity. Current guidelines advocate stress echocardiography in symptomatic patients without significant resting or provocable gradients (Class I recommendation) and asymptomatic individuals (Class IIa recommendation) to elucidate dynamic outflow obstruction [2, 5]. Prognostically, this is significant as patients with provoked obstruction are predicted to progress in their New York Heart Association (NYHA) classification from class I/II to class III at a rate of 3% per year [5]. Therefore, serial stress echocardiographic evaluation facilitates longitudinal disease trajectory and exercise tolerance assessment, informing therapeutic decision-making regarding septal reduction interventions and functional status monitoring [5, 36].

Additionally, echocardiography can visualize causes of obstruction, such as SAM of the MV or abnormal insertion of the papillary muscles. Moreover, it aids in evaluating the extent of functional MR, identifying the presence of an apical aneurysm, and quantifying diastolic dysfunction [5, 8, 20]. Thus, comprehensive echocardiographic evaluation provides essential morphologic, hemodynamic, and functional data that guide therapeutic interventions and prognostic stratification.

#### 3.2.3 Cardiac Magnetic Resonance Imaging

CMR provides a comprehensive evaluation of HCM. It is often recommended as a Class I indication for patients with HCM who have technically limited echocardiographic views or in whom the diagnosis is inconclusive via TTE [2, 45, 46]. The diagnostic criteria for HCM on CMR are similar to those used in echocardiography [5]. However, its advantage comes from its excellent spatial and temporal resolution and blood pool/myocardium contrast, which overcomes the limitations of TTE [46, 47]. CMR has particularly proven valuable in identifying areas of hypertrophy not reliably detected by echocardiography, such as the anterolateral free wall, apex, or posterior septum. This is especially important as roughly 20% of patients have focal HCM in one or two LV segments [46, 47]. Moreover, even in patients in whom apical HCM was detected on echocardiography, there was a significant discrepancy between the measured apical wall thickness on TTE vs CMR (mean difference of 1.7 mm) [48]. TTE also overestimates LV wall thickness in 59% of patients due to off-axis imaging, right ventricular (RV) trabeculations, or papillary muscle inclusion [48]. With TTE being operator and reader-dependent, it is no surprise that a study found a significantly lower interobserver variability with CMR than TTE [49]. Lastly, CMR can more accurately identify high-risk features such as the presence of LV apical aneurysm, severe LVH (≥30 mm), extensive late gadolinium enhancement (LGE) of ≥15% irrespective of location or pattern in the LV wall, and LV ejection fraction (LVEF) of <50%, all of which pose an increased risk of SCD from lethal ventricular arrhythmias [2, 5].

LVH is a non-specific finding frequently observed on TTE, making it challenging to differentiate HCM from its phenotypic mimics. However, CMR can be used to distinguish an athlete’s heart from HCM via the identification of focal hypertrophy (in favor of HCM) or regression in maximal LV wall thickness by ≥2 mm after a period of systemic deconditioning (in favor of an athlete’s heart) [47]. Beyond morphologic assessment, tissue characterization with LGE has become a hallmark of CMR evaluation. For example, LGE can identify focal areas of replacement fibrosis, which are present in up to 70% of HCM patients, a finding typically absent in an athlete’s heart [47, 50].

Infiltrative cardiomyopathies that phenotypically simulate HCM demonstrate distinctive CMR signatures: cardiac amyloidosis manifests as subendocardial and transmural enhancement with relative apical sparing and characteristic simultaneous myocardial and blood nulling; Fabry disease exhibits enhancement of mid-lateral wall segments with subendocardial sparing; and Danon disease presents with severe LVH accompanied by extensive enhancement with mid-septal sparing [20]. These pathognomonic enhancement patterns provide critical diagnostic information that guides therapeutic interventions, facilitates familial genetic counseling, and enables longitudinal assessment of disease progression.

#### 3.2.4 Cardiac Catheterization and Cardiac Computed Tomography Angiography

While non-invasive modalities remain the cornerstone of hemodynamic characterization in HCM, invasive cardiac catheterization assumes an essential diagnostic role in clinical scenarios where echocardiographic data proves inconclusive, technically inadequate, or is contraindicated [2, 51]. Specific indications include circumstances where Doppler echocardiography cannot differentiate between an increase in velocity profile from an LVOTO versus contamination by MR, necessitating direct pressure gradient measurement with corresponding waveform analysis [2, 51]. Additional indications for invasive hemodynamic assessment encompass patients with concomitant aortic stenosis and dynamic outflow obstruction, and those with symptomatic burden disproportionate to resting non-invasive imaging findings [2, 51]. Moreover, patients with persistent chest pain warrant catheterization to accurately rule out coronary artery disease (CAD) due to the high false-positive and negative rates associated with nuclear and echocardiographic stress testing [2]. Lastly, for patients planned for surgical myectomy (SM) or alcohol septal ablation (ASA), coronary angiography is often performed to aid procedural planning [2].

Since hemodynamic assessment of HCM is crucial for guiding management, the role of cardiac computed tomography angiography (CCTA) remains limited. As a result, it holds a Class IIb recommendation for assessing patients with suspected HCM if an echocardiogram is inconclusive and CMR is contraindicated [2]. However, due to its excellent three-dimensional resolution, CCTA can reveal morphological features of HCM to establish a diagnosis, evaluate for myocardial bridging, and visualize septal perforators within the myocardium [20].

#### 3.2.5 Cardiopulmonary Exercise Testing

Exercise stress testing is safe for HCM patients and provides valuable information on their functional capacity and limitations. Studies have shown that patients with reduced peak oxygen consumption (≤15.3 mL/kg/min), ventilatory efficiency (VE/V_CO2_ >34), and anaerobic threshold on cardiopulmonary exercise testing (CPET) have a higher rate of ventricular arrhythmias, progression to advanced heart failure, and a higher all-cause mortality [2, 52, 53, 54, 55, 56]. Therefore, CPET should be used as part of a standard evaluation for symptomatic patients, especially those with severe symptoms being considered for heart transplant [2].

#### 3.2.6 Endomyocardial Biopsy

Endomyocardial biopsy plays a crucial role in diagnosing HCM, especially in cases where non-invasive imaging and genetic testing do not yield definitive results. Histologically, HCM is characterized by myocyte hypertrophy with enlarged, hyperchromatic nuclei, disorganized myofiber architecture known as myofiber disarray, and interstitial fibrosis marked by increased collagen deposition [57, 58, 59]. In contrast, amyloidosis, an infiltrative cardiomyopathy, can mimic HCM on imaging but has distinct histopathological features: extracellular amyloid deposits which stain positively with Congo red and exhibit apple-green birefringence under polarized light [60]. These deposits appear as rigid, non-branching fibrils of 7–10 nm diameter on electron microscopy (EM) [61], and unlike the patchy fibrosis with myocyte disarray in HCM, amyloid infiltration results in concentric myocardial thickening. Identifying these differences is critical for appropriate treatment and prognosis [62, 63].

EM offers additional diagnostic precision by revealing ultrastructural abnormalities that are not visible via light microscopy [64]. Common EM findings include variability in mitochondrial size, swelling, cristae disruption, paracrystalline inclusions, and distinctive crystalline structures within mitochondria that suggest mitochondrial dysfunction [62]. These features help differentiate primary HCM from phenocopies caused by metabolic or lysosomal storage diseases such as Fabry disease, where EM detects characteristic lamellar inclusions [62, 65]. Such ultrastructural insights are valuable because they augment the pathological understanding and tailor patient management.

## 4. Screening 

Contemporary screening guidelines for relatives of HCM probands have been meticulously delineated in the 2024 American College of Cardiology/American Heart Association (ACC/AHA) consensus recommendations [2]. Cascade genetic testing is indicated for first-degree relatives when a pathogenic (P) or likely pathogenic (LP) sarcomeric variant has been identified in the proband; conversely, in genotype-negative HCM probands, familial genetic screening yields limited diagnostic utility, although phenotypic surveillance remains imperative [2]. The screening protocol involves comprehensive risk stratification for SCD and AF and objective assessment of functional capacity [2]. Initial testing involves ECG, TTE, and genetic testing. However, additional testing for HCM mimics (PRKAG2, LAMP2, GLA, etc.) should also be performed if the affected patient meets specific disease phenotypes [2, 20, 44]. American and European guidelines vary with regard to the time of genetic testing in the pediatric population. European guidelines suggest screening after the age of 10–12 years, whereas American guidelines suggest screening children and adolescents at the time a pathogenic variant of HCM is diagnosed in a family member [2, 66]. In this population, TTE and ECG should be repeated every 1–2 years [2, 20]. For pediatric patients in whom family history is negative for a pathogenic variant, screening is recommended at any time after the family member was diagnosed, but no later than puberty. TTE and ECG should be repeated in this population every 2–3 years [2, 20].

Screening for adult family members of a proband depends on the phenotype of the individual being tested. In HCM phenotype-positive adults, a baseline evaluation includes SCD risk assessment by TTE, stress echocardiogram, ECG, ambulatory ECG (to rule out AF), and CMR (to evaluate for LGE and apical aneurysm). Repeat clinical assessment, TTE, and ambulatory ECG monitor should be performed in these patients every 1–2 years [2]. Stress TTE or CPET should be considered in asymptomatic adults every 2–3 years to assess for occult serial decline in functional status [2]. In symptomatic adults, it is crucial to assess for worsening dynamic LVOTO every 1–2 years via stress TTE (if their resting gradient is <50 mmHg), or by CPET if they have a significant decline in functional status or are being considered for advanced heart failure (HF) therapies [2, 20, 35]. CMR may be helpful in the continuous assessment of HCM, and is recommended every 3–5 years, assuming no implantable cardioverter defibrillator (ICD) is present already, to assess for apical aneurysms or worsening LGE [2]. It is also important to note that the follow-up testing intervals may be extended if a patient demonstrates stability over multiple visits.

In adults with phenotype-negative HCM, screening intervals depend on the presence of a P/LP genotypic variant in the patient and family. For patients who don’t have a P/LP variant or have a family and personal history of a P/LP variant, it is recommended that they undergo screening with ECG and TTE (CMR if TTE is insufficient) every 3–5 years [2, 20]. Conversely, if a patient has a known family history of a P/LP variant but has no variant on genetic testing, no additional surveillance is required. Table 2 (Ref. [2, 5]) summarizes the screening recommendations adapted from the 2024 ACC/AHA HCM Guideline.

**Table 2.** **Screening guidelines for family members of a proband with hypertrophic cardiomyopathy**.

	Pediatric population	Adult population
Initiation of screening [2, 5]	• If genotype-positive family, or early-onset disease in family, screening starts at the time of HCM diagnosis in the proband (preferably after 12 years of age)	• At the time of HCM diagnosis in another family member
		• Continued surveillance from adolescent screening protocols upon transition to adult care
Screening Intervals [2, 5]	• Genotype-positive/early-onset disease families: Every 1–2 years (until 18–21 years of age)	• In phenotype-negative patients: Every 3–5 years
	• All other pediatric cohorts: Every 2–3 years (until 18–21 years of age)	• In phenotype-positive patients: Every 1–2 years with standard diagnostic testing ± CMR every 3–5 years
Imaging modalities [2, 5]	• Initial approach: Baseline 12-lead ECG, ambulatory ECG, TTE, stress TTE (to assess for dynamic obstruction)	• Initial approach: Baseline 12-lead ECG, ambulatory ECG, TTE, stress TTE (to assess for dynamic obstruction)
	• CMR when echocardiographic findings are inconclusive	• ECG, ambulatory ECG, and TTE every 1–2 years
		• CMR every 3–5 years for SCD risk assessment in absence of ICD
		• Exercise testing for functional status assessment every 2–3 years in asymptomatic individuals
Symptomatic individuals [2, 5]	• Exercise testing with stress TTE (if LVOT gradient <50 mmHg)	
	• CPET for functional status assessment and consideration of advanced heart failure therapies	
Genetic testing [2, 5]	• Recommended for family members of a patient with a pathogenic variant	
	• In atypical clinical presentations of HCM, genetic testing for HCM and HCM phenocopies should be performed	
	• Unclear usefulness in assessing the risk of SCD	
	• Cascade genetic testing is not recommended for relatives of a proband without a pathogenic variant	

Table describing the screening guidelines for family members of a relative with HCM, adapted from the 2024 ACC/AHA guidelines. HCM, hypertrophic cardiomyopathy; ACC/AHA, American College of Cardiology/American Heart Association; CMR, cardiac magnetic resonance imaging; ECG, electrocardiogram; TTE, transthoracic echocardiography; SCD, sudden cardiac death; ICD, implantable cardioverter defibrillator; LVOT, left ventricular outflow tract.

## 5. Sudden Cardiac Death

### 5.1 Risk Factors 

**Fig. 2.**
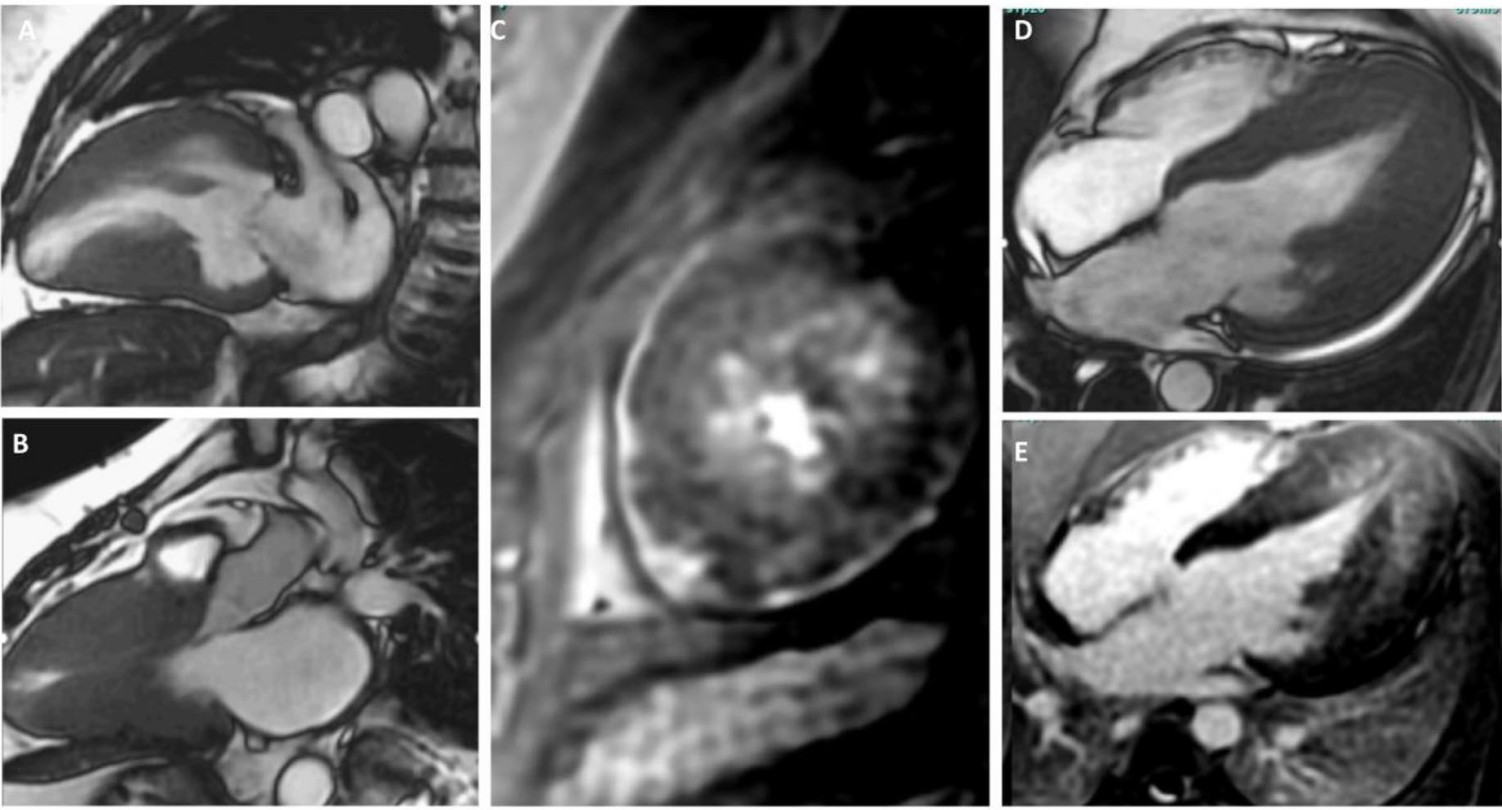
**Cardiac MRI Cine SSFP images showing 2,3, and 4-chamber views highlighting additional characteristics seen in hypertrophic cardiomyopathy**. (A,B,D) showcases severely hypertrophied walls. (A,B) showcase mid-ventricular hypertrophy with an aneurysmal apex (A). (E) showcases apical hypertrophy with mid-wall late gadolinium enhancement. (C) shows a short-axis view with mid-wall late gadolinium enhancement of the left ventricular apex. MRI, magnetic resonance imaging; SSFP, steady-state free precession.

**Fig. 3.**
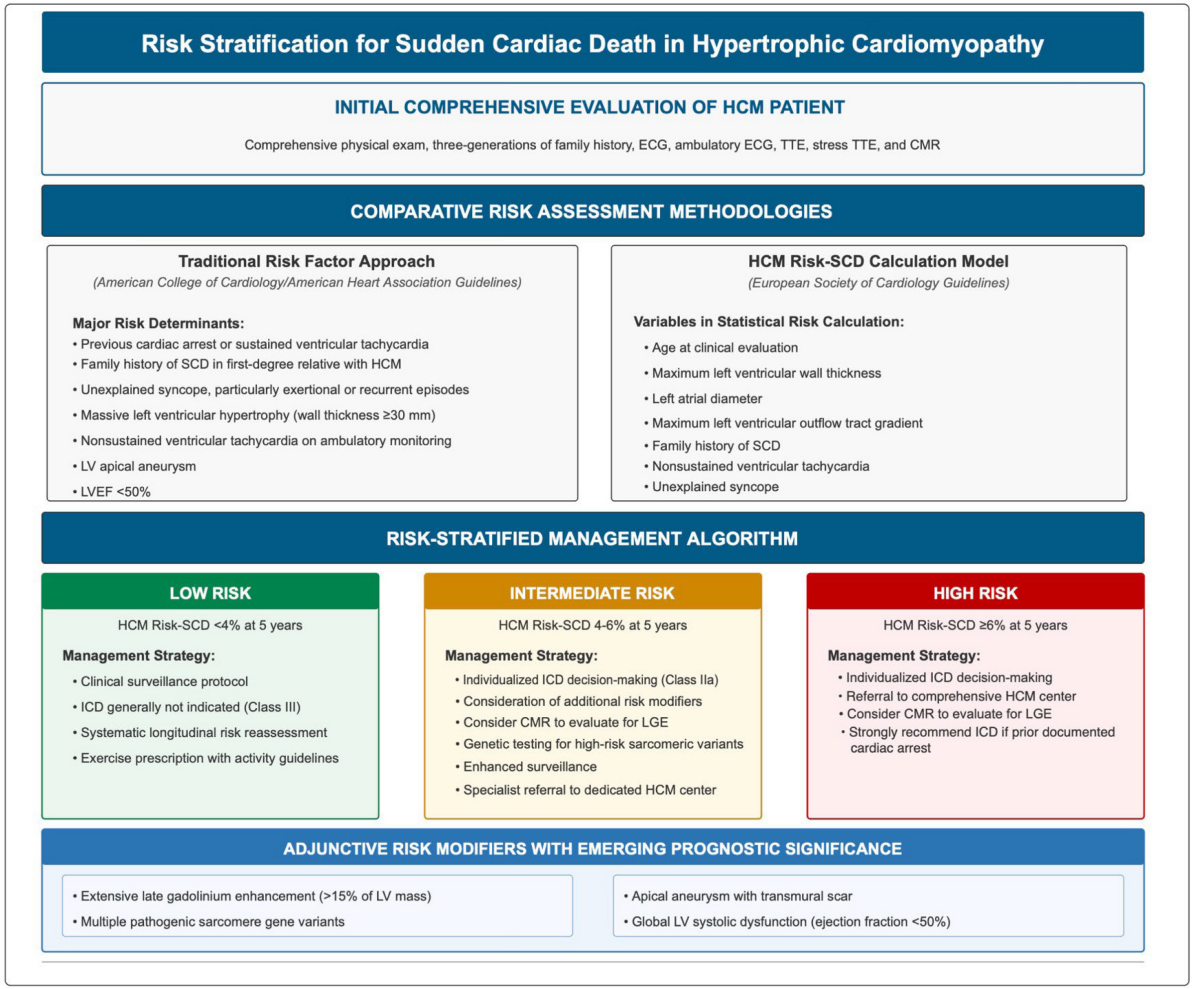
**Risk stratification for sudden cardiac death in hypertrophic cardiomyopathy [74, 75]**. HCM, hypertrophic cardiomyopathy; CMR, cardiac magnetic resonance imaging; ECG, electrocardiogram; TTE, transthoracic echocardiography; SCD, sudden cardiac death; ICD, implantable cardioverter defibrillator; LV, left ventricular; LVEF, left ventricular ejection fraction; LGE, late gadolinium enhancement.

The overall risk of SCD in HCM is relatively low and is estimated to be 0.5% per year [20]. However, the risk of SCD should be assessed based on the individual patient. For example, in a study of SCD among athletes of various ages, the sports with the highest incidence of SCD include basketball, football, and soccer. Moreover, a higher incidence rate was noted in Black male NCAA Division I college athletes compared to their high school counterparts [67, 68]. Risk stratification for SCD is a critical component in managing patients with HCM and has evolved considerably over the past several decades. Multiple clinical risk markers have been identified that stratify patients according to their level of risk for potentially life-threatening ventricular arrhythmias. These established risk factors include: family history of SCD in first-degree or close relatives ≤50 years of age; massive LVH (wall thickness ≥30 mm in any segment); unexplained syncope, particularly when occurring within 6 months of evaluation; LVEF <50%; presence of apical aneurysm with transmural scar (Fig. 2, Video 2); extensive LGE on CMR (≥15% of LV mass); and non-sustained ventricular tachycardia (NSVT) on ambulatory monitoring. Based on the 2024 AHA/ACC HCM Guideline, the presence of ≥1 of these major features may justify ICD placement for primary prevention (Class IIa recommendation) [2, 69]. Increased LA diameter has also been shown to correlate with an increase in the risk of SCD in one study, though evidence from further studies has been controversial [2, 20, 70]. Nevertheless, it has been added to the HCM risk-SCD calculator for estimating the 5-year risk of SCD, along with the LVOT gradient. However, the SCD risk estimate does not account for the newer factors (LVEF <50%, LGE, apical aneurysm) as their impact on the 5-year risk estimate is currently undetermined [2]. A growing body of literature demonstrates a consistent correlation between these factors and the resultant increase in risk of SCD, making it more likely for these findings to be integrated into a new scoring system [71, 72, 73]. Fig. 3 (Ref. [74, 75]) summarizes the risk stratification guidelines for SCD in HCM.

**Video. 2.** **Cardiac magnetic resonance imaging highlighting apical aneurysm in hypertrophic cardiomyopathy**. Video associated with this article can be found, in the online version, at https://doi.org/10.31083/RCM42824.

Despite the current guidelines, there remains conflicting evidence regarding screening criteria for genotype-positive, phenotype-negative individuals, given the phenotypically heterogeneous nature of HCM. Family members with identical mutations have been shown to display different phenotypic expression, which indicates the potential role of exogenous factors in disease development [76]. There is evidence that these patients have impaired myocardial relaxation and impaired energy metabolism, but the clinical implication of these findings on the disease onset or severity and the risk of sudden cardiac death remains unknown, which makes screening and treatment extremely challenging [76].

### 5.2 Implantable Cardioverter Defibrillator Guidelines 

ICD therapy is an effective intervention for the primary prevention of lethal ventricular arrhythmias and SCD. However, due to the complexities associated with HCM, not all patients benefit from this treatment [77].

### 5.3 Device Considerations

ICD selection has evolved from advocating “all-purpose” ICD systems toward a nuanced approach predicated on individualized patient characteristics and specific clinical scenarios. Single-chamber devices and subcutaneous ICDs (S-ICDs) represent reasonable options for most HCM patients without pacing requirements [78].

S-ICDs offer particular advantages for young patients with extended life expectancy who face a heightened cumulative risk of complications related to transvenous leads. Studies from the EFFORTLESS cohort illustrate these benefits, indicating a negligible risk of bloodstream infections, a low incidence of lead fractures, and an impressive 2-year estimate of 92.7% for freedom from procedural complications compared to traditional transvenous ICDs [78]. Furthermore, findings from the PRAETORIAN trial have shown that S-ICDs are non-inferior to transvenous ICDs in terms of inappropriate shocks among general ICD candidates. As a result, S-ICDs have gained popularity among patients with HCM without additional requirements [78, 79].

The nuanced approach stems from patient-specific clinical scenarios that warrant specific device selection, such as:

1. Left ventricular outflow tract obstruction: Transvenous systems may be preferred for oHCM patients, particularly if septal reduction procedures might be required, as these interventions carry a non-negligible risk of conduction defects (10% in ASA patients, 4.4% in SM patients) necessitating pacing capabilities [80]. Moreover, changes in QRS-T morphologies after septal reduction therapies (SRT) may result in T-wave oversensing and failure to recognize the stored QRS-T template, leading to inappropriate shocks [78]. Therefore, patients with oHCM who may require SRT in the future may benefit from transvenous ICD, rather than S-ICD, therapy upfront.

2. End-stage disease: Patients who develop “burnt out” HCM, characterized by advanced heart failure with reduced ejection fraction, face an annual event rate of 10% for life-threatening arrhythmias [78]. Given that left bundle branch block (LBBB) is prevalent in such cases, cardiac resynchronization therapy with a defibrillator (CRT-D) may offer more advantages than S-ICDs. This is especially relevant in patients exhibiting severe interventricular septal fibrosis on CMR, as this condition heightens concerns about potential heart block that may necessitate pacing [78].

3. Pediatric patients: High complication rates (approximately 9.5% per year) have been documented in children and adolescents with HCM, primarily involving inappropriate shocks (41%), lead malfunction, and lead stretching [78, 81]. As a result, S-ICDs may be a better alternative in this population. However, a significant limitation is their large size in relation to the transvenous ICD and the body size of the child (due to concern for device erosion in smaller patients) [2, 78, 81].

## 6. Pharmacologic Management

### 6.1 Beta-Blockers and Calcium Channel Blockers

Non-vasodilating beta-blockers (BBs) are regarded as first-line therapy for symptomatic oHCM [2, 35, 82]. They work by increasing LV filling time and volume (negative chronotropy) while decreasing the contractile force (negative inotropy), attenuating dynamic LVOTO and mitigating associated symptoms [2, 35, 82]. Although these drugs are widely used, there is a surprising lack of studies assessing their effectiveness, with the majority being underpowered and not randomized [82]. For example, all the studies on BBs found significant variability in LVOT gradient reduction, limited improvement in symptoms, and a stark proportion of non-responders, raising the need for larger randomized trials to better understand the role of these medications, especially in non-responders [82]. BB dosing is individualized, with medications uptitrated until a patient achieves symptomatic relief. However, if patients continue to have suboptimal response despite maximum dosing, non-dihydropyridine CCBs, such as verapamil or diltiazem, can be substituted [2, 35, 82]. CCBs also work by providing negative inotropic and chronotropic effects; however, they pose the risk of exacerbating outflow gradients in some patients. In fact, verapamil is specifically contraindicated for patients with severely elevated resting gradients (>100 mmHg), hypotension, and severe dyspnea at rest [2, 82].

Regarding treating asymptomatic carriers, it is crucial to continue close clinical surveillance to monitor for symptom onset or for the presence of additional risk factors, which might warrant further management. Pharmacotherapy should be utilized to provide symptomatic relief and improve quality of life, but the role of medical therapy in asymptomatic carriers is currently not recommended and remains a topic of ongoing investigation. Overmedicalization of asymptomatic carriers poses an additional financial and psychosocial burden on these patients.

### 6.2 Disopyramide

Disopyramide serves as an additional treatment option for patients with oHCM who remain symptomatic despite first-line pharmacotherapy [2]. Because it can enhance conduction through the atrioventricular (AV) node, potentially facilitating rapid ventricular rates during AF, disopyramide should only be administered with BBs or CCBs [2].

As a class I antiarrhythmic agent, disopyramide possesses potent negative inotropic effects. Recent studies have demonstrated that it influences multiple ion channels, reduces the inward calcium current, and stabilizes the ryanodine receptor. This stabilization subsequently leads to diminished calcium release from the sarcoplasmic reticulum. Furthermore, disopyramide inhibits the late inward sodium current, which is often elevated in HCM and contributes to early and late afterdepolarizations. This mechanism may help mitigate the risk of ventricular arrhythmias in these patients [83, 84].

**Fig. 4.**
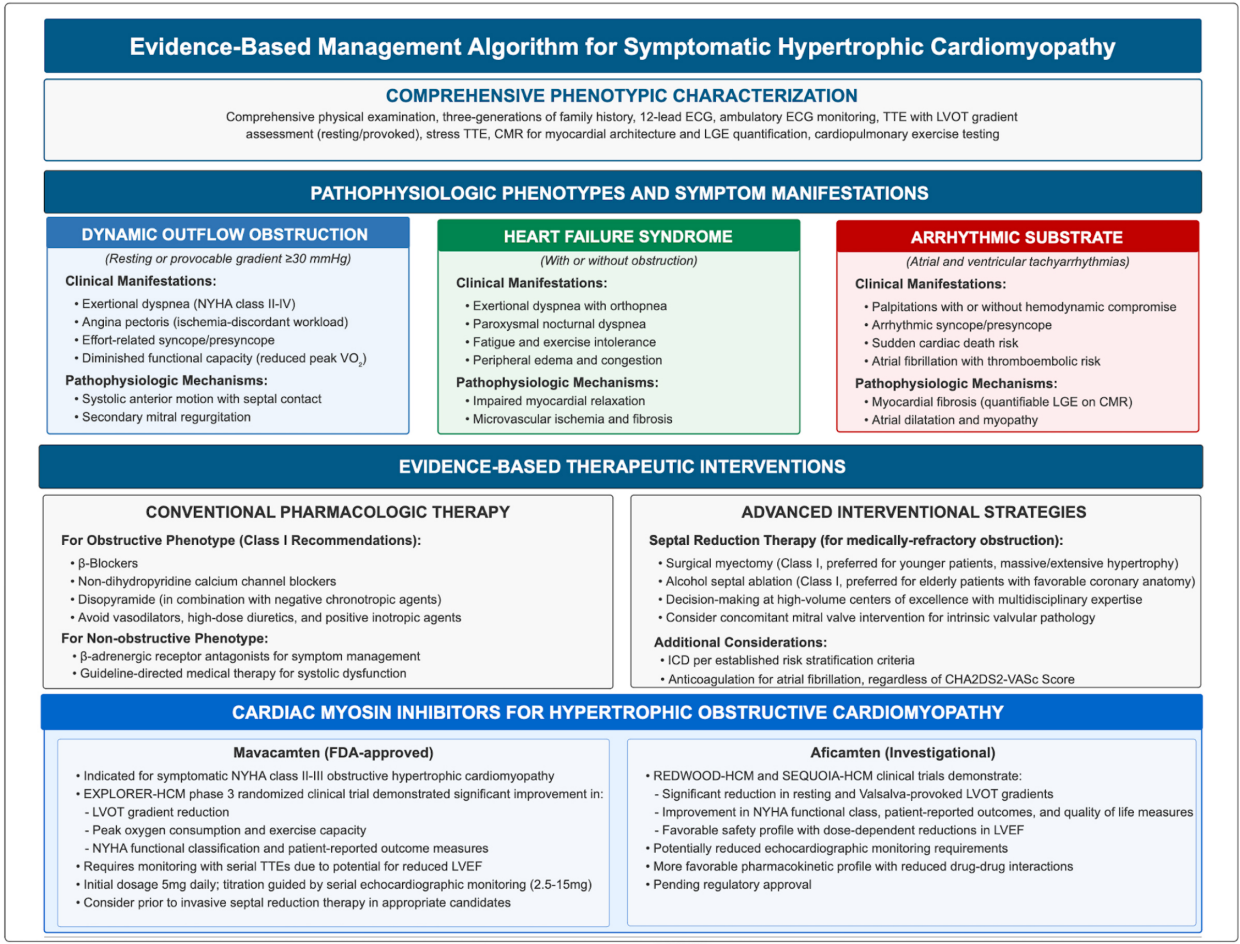
**Evidence-based management algorithm for symptomatic hypertrophic cardiomyopathy [2, 85, 86, 87, 88, 89, 90]**. HCM, hypertrophic cardiomyopathy; CMR, cardiac magnetic resonance imaging; ECG, electrocardiogram; TTE, transthoracic echocardiography; LVEF, left ventricular ejection fraction; LVOT, left ventricular outflow tract; NYHA, New York Heart Association; FDA, Food & Drug Administration; LGE, late gadolinium enhancement.

Disopyramide’s cost-effectiveness allows better access for patients of lower socioeconomic backgrounds, unlike the significantly more expensive mavacamten discussed in section 7.3 [76, 77, 78]. Fig. 4 (Ref. [2, 85, 86, 87, 88, 89, 90]) summarizes the overall management of patients with symptomatic HCM.

### 6.3 Mavacamten

Mavacamten is a first-in-class reversible cardiac myosin inhibitor that, unlike negative inotropes, exhibits a novel mechanism of action through selective modulation of the sarcomeric contractile apparatus [91].

At the molecular level, mavacamten acts as an inhibitor of the β-cardiac myosin adenosine triphosphatase (ATPase). ATP typically binds to the myosin head and is hydrolyzed by ATPase into adenosine diphosphate (ADP), allowing the myosin head to form a cross-bridge with actin and initiate a power stroke [85, 91]. By inhibiting ATPase, mavacamten increases the number of myosin heads in a relaxed, energy-sparing state (ATP bound) while decreasing the number of myosin heads in a power-generating state (ADP bound). As a result, it reduces cardiac hypercontractility by lowering the frequency of actin-myosin cross-bridge formation [85, 91].

In the landmark phase 3 EXPLORER-HCM randomized controlled trial, mavacamten demonstrated significant efficacy in HCM patients with LVOT obstruction. This pivotal trial enrolled 251 patients with symptomatic oHCM (NYHA class II–III, and LVOT gradient ≥50 mmHg at rest or with provocation) randomized to mavacamten or placebo [86]. After 30 weeks of treatment, mavacamten produced a remarkable improvement through an increase in peak oxygen consumption (pVO_2_) by ≥1.5 mL/kg/min along with a ≥1 reduction in NYHA class, or ≥3 mL/kg/min increase in pVO_2_ without worsening of NYHA class in 37% of patients [85, 86]. Finally, long-term extension data from the EXPLORER-HCM study (MAVA-LTE) revealed a substantial improvement in LVOT gradients, achieving non-obstructive levels in 82.7% of patients. Furthermore, by the conclusion of the 3.5-year period, 67.4% of patients had returned to NYHA I status [87].

Since the discovery of mavacamten, several trials have been conducted to elucidate its benefits across various patient populations. The VALOR-HCM study enrolled 112 patients who fit the criteria for septal reduction therapy, defined by an LVOT gradient of ≥50 mmHg at rest or during provocation. These patients were randomized in a double-blind manner to receive either a placebo or mavacamten, which was titrated from a starting dose of 5 mg to a maximum dose of 15 mg daily. The study’s primary endpoint was the proportion of patients who either proceeded with septal reduction therapy or remained eligible for it according to guidelines after 16 weeks of treatment. At the conclusion of the study, only 17.9% (10 out of 56) of the mavacamten patients underwent septal reduction therapy, compared to 76.8% (43 out of 56) of those in the placebo group. Long-term data further supported the effectiveness of mavacamten, as 89% (96 out of 108) of patients continued to take the medication without needing septal reduction therapy. This suggests that mavacamten can reduce the need for surgery through medical management alone [92, 93]. Additional exploratory analysis from this study also revealed similar and sustained longitudinal benefits from mavacamten in all patients irrespective of genotype status [94].

The EXPLORER-CN study randomized 81 Chinese patients with oHCM to mavacamten or placebo and found a significant improvement in Valsalva LVOT gradient in the mavacamten arm [95].

**Fig. 5.**
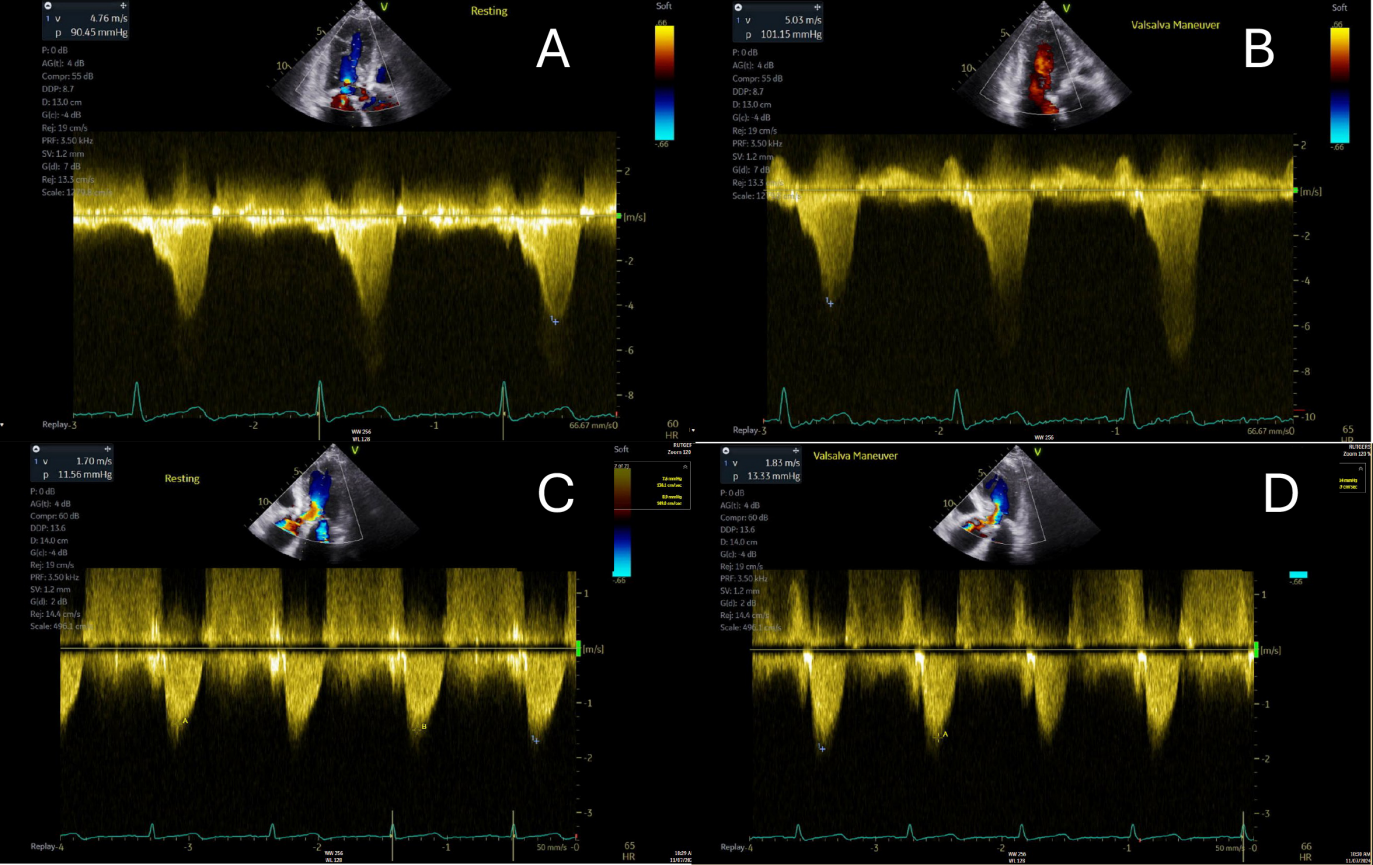
**Pre- and post-mavacamten left ventricular outflow tract gradients in the same patient with hypertrophic cardiomyopathy are illustrated**. (A,B) depict the pre-mavacamten gradients at rest (A) and during Valsalva maneuver (B). (C,D) present the post-mavacamten gradients at rest (C) and with Valsalva (D). Notably, there is a significant improvement post-mavacamten, with no provokable gradient observed during the Valsalva maneuver.

It should be noted that the effects of mavacamten have only been found to be beneficial in HCM patients with LVOTO, as major landmark trials (i.e., EXPLORER-HCM, VALOR-HCM) have specifically included LVOTO patients and excluded those with mid-ventricular obstruction. As discussed in section 10, the ODYSSEY-HCM trial sought to address the role of mavacamten in non-obstructive HCM patients (including those with mid-ventricular obstruction or apical HCM). However, a recent update from the study investigators reveals no significant improvement in peak oxygen consumption or patient-reported quality of life [96, 97, 98]. Altogether, these trials showcase the significant symptomatic and hemodynamic benefits of mavacamten in patients with LVOTO and underscore its potential to provide a better quality of life for HCM patients. Fig. 5 showcases the effects of mavacamten on the LVOT gradient in the same patient pre- and post-therapy.

The principal safety concern with mavacamten is its negative inotropic effect, manifesting as a reduction in LVEF. In the EXPLORER-HCM trial, 7 patients (5.4%) receiving mavacamten experienced LVEF <50%, necessitating temporary dose reduction or treatment interruption, with all cases resolving after protocol-directed interventions [86]. Therefore, the 2024 ACC/AHA HCM Guideline recommends discontinuing mavacamten for patients who develop an LVEF of <50% and fail to recover [2]. Hence, rigorous echocardiographic surveillance should be performed, particularly during dose titration and in patients with borderline systolic function. The United States Food & Drug Administration (FDA) recommends obtaining a TTE every 4 weeks for the first 12 weeks after initiating mavacamten. After this initial period, routine TTEs should be performed every 6 months per recent updates, assuming the LVEF and LVOT gradients remain stable (LVEF ≥55% and LVOT gradient <30 mmHg) [99].

If treatment is halted due to LVEF dropping below 50%, it is recommended to repeat TTEs every 4 weeks until LVEF exceeds 50%. Once LVEF is stable, treatment can be restarted at half the previous dose, with an uptitration after two TTEs obtained 4 weeks apart from each other show a stable LVEF [99].

### 6.4 Aficamten 

Aficamten, like mavacamten, is a selective cardiac myosin inhibitor that modulates sarcomere function by reducing actin-myosin cross-bridge formation [88]. However, there are key differences between the two. Aficamten was specifically designed with a shallow dose-response curve, meaning that increases in dosage lead to only modest reductions in LVEF. This feature provides it with a broader therapeutic window compared to mavacamten [88]. Additionally, aficamten has a shorter half-life of 3.4 days, whereas mavacamten has a half-life of 7 to 9 days. This shorter half-life facilitates more rapid titration and allows for quicker reversibility after dose adjustments [85].

The SEQUOIA-HCM trial was the first major landmark trial to explore the efficacy of aficamten among 142 of the 282 patients enrolled with symptomatic oHCM across multiple clinically relevant endpoints [88]. At the 6-month mark, 58.5% of patients had improvement in their baseline NYHA class by ≥1, 49.3% had an LVOT gradient of <30 mmHg with a rapid reduction of at least 20 mmHg in the first 2 weeks, and 47% of patients had a significant improvement in exercise capacity—even in severely symptomatic NYHA III or IV patients [88, 89]. Following a 4-week washout period, patients returned to their baseline echocardiographic parameters but experienced a worsening of their health status, further demonstrating rapid pharmacologic reversal of the drug and potentially creating the problem of medication reliance in the future [100]. Lastly, in contrast to the EXPLORER-HCM trial which suggested a reduction in the benefits of mavacamten in patients taking BBs, the benefits of aficamten were similar regardless of BB use and independent of the presence of a pathogenic sarcomere gene variant [88]. Additional head-to-head studies are required to compare mavacamten and aficamten in a long-term setting to determine which generation of myosin inhibitors is superior.

## 7. Invasive Management 

SRTs are definitive interventions for patients with oHCM who remain symptomatic despite maximal tolerated medical therapy. These procedures should be conducted in comprehensive HCM centers (2024 ACC/AHA HCM Guideline, Class I recommendation) for optimal patient outcomes [2]. The two main types of SRT are SM and ASA.

### 7.1 Septal Myectomy 

**Fig. 6.**
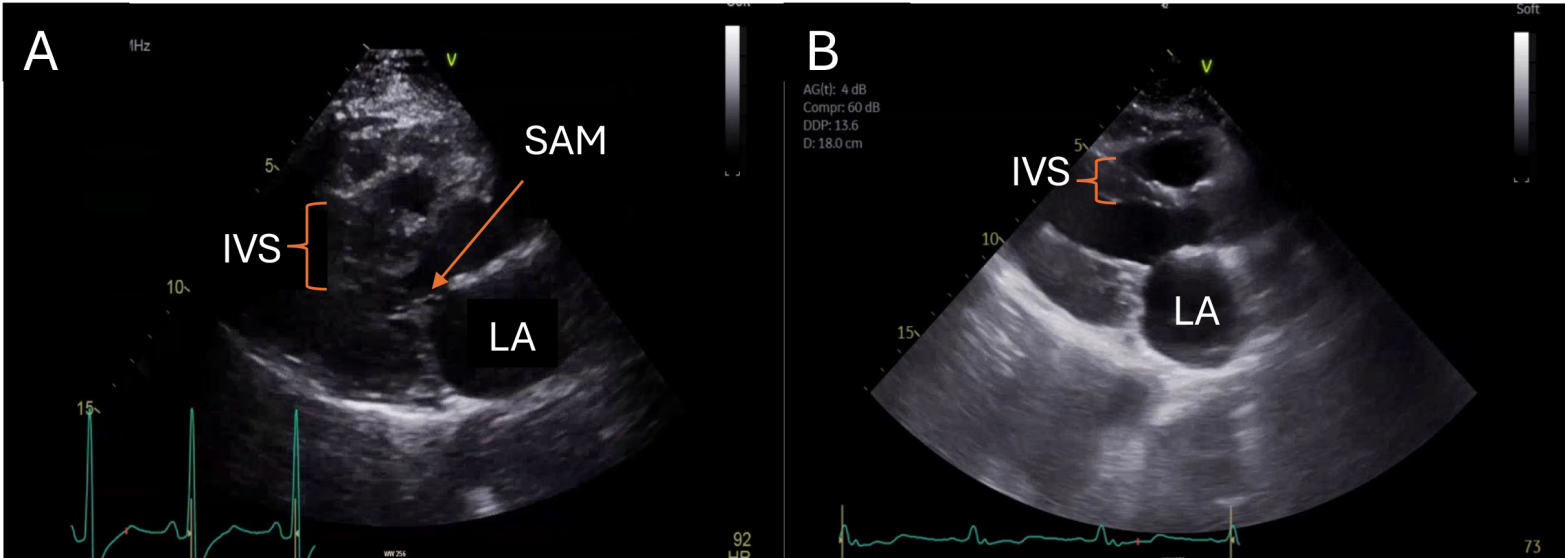
**Parasternal long-axis views of a patient pre- and post-myectomy are illustrated**. Systolic anterior motion of the anterior mitral valve leaflet in the setting of marked basal hypertrophy in the patient pre-myectomy (A) is no longer observed after myectomy (B). IVS, interventricular septum; SAM, systolic anterior motion; LA, left atrium.

SM is an open-heart intervention characterized by median sternotomy, cardiopulmonary bypass, aortic cross-clamping, and a transaortic approach for the excision of a segment of the IVS to alleviate the LVOTO [101, 102]. The classic Morrow procedure, introduced in the 1960s, has evolved from a straightforward excision of the basal IVS from the aortic valve annulus to a more extensive septal excision. This modern technique extends well beyond the mitral-septal contact point and involves the midventricular septum, reaching up to the level of the papillary muscles [101]. Myectomy, therefore, reliably results in the immediate and typically permanent elimination of outflow obstruction, with normalization of LV pressures and preservation of systolic function via the enlargement of the LVOT cross-sectional area and subsequent redirection of blood flow away from the anteriorly displaced MV seen in SAM. Fig. 6 and Video 3A,3B showcase the immediate results of a patient’s outflow tract gradient pre- and post-myectomy. During SM, a comprehensive evaluation via intra-, and post-operative transesophageal echocardiogram (TEE) may be done to assess the hemodynamic implications of the procedure, including the persistence of the LVOT gradient. Should such gradients remain problematic, re-establishment of cardiopulmonary bypass may be warranted to facilitate a more extensive myectomy [101].

**Video. 3A.** **Systolic Anterior Motion Pre- and Post-myectomy in HCM**. Video 3A (still image) showcases SAM and outflow tract obstruction in a patient pre-myectomy. HCM, hypertrophic cardiomyopathy; SAM, systolic anterior motion. Videos associated with this article can be found, in the online version, at https://doi.org/10.31083/RCM42824.

**Video. 3B.** **Systolic Anterior Motion Pre- and Post-myectomy in HCM**. Video 3B (still image) showcases post-myectomy resolution of SAM and relief of outflow tract obstruction. HCM, hypertrophic cardiomyopathy; SAM, systolic anterior motion. Videos associated with this article can be found, in the online version, at https://doi.org/10.31083/RCM42824.

Optimal candidates for SM include symptomatic (NYHA III–IV) patients with oHCM who have additional structural diseases that require intervention. These conditions may include anomalous insertion of the papillary muscle, severe elongation of the anterior or posterior mitral leaflet (greater than 30 mm), multivessel CAD, and valvular aortic stenosis [2, 101]. Additionally, patients with AF who might benefit from intraoperative pulmonary vein isolation or a maze procedure should also be considered for SM [2]. NYHA class II patients with significant pulmonary hypertension secondary to LVOTO or associated MR, poor exercise capacity, and children and young adults with severely elevated LVOT gradients of >100 mmHg should also be considered for SM in highly experienced centers (Class IIb level of evidence per 2024 ACC/AHA HCM Guideline) [2]. Lastly, while it may be tempting to replace the mitral valve for MR during SM, studies have shown that valve replacement, as opposed to valve repair or a valve-sparing approach, leads to a >10-fold increase in hospital mortality and length of stay compared to isolated SM [2, 18, 103].

In general, SM, when done at comprehensive HCM centers, carries exceptional success rates of >90% to 95%, with an overall estimated procedural mortality of <1% [2, 104]. However, it is not without its complications. Conduction abnormalities are common in patients after SM, with a 38% risk of developing an LBBB and a 2.3% risk of developing complete heart block (CHB) [105]. On the other hand, the risks of ventricular septal defect (VSD), aortic regurgitation (AR), ventricular arrhythmias, cardiac tamponade, and stroke were found to be <1% in the modern era [104, 106].

The importance of performing SM at comprehensive and highly skilled HCM centers is underscored when comparing the outcomes of these specialized centers with those of lower-volume facilities. For instance, data from the Mayo Clinic, which analyzed over 3000 SM procedures conducted between 1993 and 2016, revealed impressive results. Specifically, there was less than a 1% risk of post-operative death, a 2% chance of CHB that required the insertion of a pacemaker, and an iatrogenic risk of ventricular septal defect of 0.3% [107]. Additionally, 90% of severely symptomatic patients experienced an improvement of more than 2 NYHA classes, leading to a significant relief from their symptoms [107].

Similarly, Tufts Medical Center reported favorable outcomes for 482 SM procedures performed from 2003 to 2016. They recorded a post-operative mortality rate of 0.8%, with 94% of patients improving to NYHA Class I or II, owing to substantial reductions in LVOT gradients and a decrease in associated mitral regurgitation [108].

In contrast, a recent study from the National Readmission Database, which tracked patients receiving septal reduction therapy from 2010 to 2019, found that those who underwent SM at low-volume centers faced double the risk of complications, such as needing a pacemaker (11.8% compared to 6.9%). These patients also had higher rates of composite in-hospital mortality and 30-day readmissions (21.7% versus 11.8%) [109].

The significant disparities between low- and high-volume HCM centers highlight the critical need to refer patients to specialized institutions to ensure better outcomes.

### 7.2 Alcohol Septal Ablation

ASA is a therapeutic option for adult patients with symptomatic oHCM refractory to maximal pharmacological intervention, particularly in those for whom surgical intervention is contraindicated or confers prohibitive risk due to significant comorbidities or advanced age [2]. This percutaneous interventional procedure involves the targeted delivery of alcohol via a catheter-based approach to a selectively engaged septal perforator artery, inducing a precisely controlled septal myocardial infarction [110]. Appropriate targets for ASA are determined by several factors, including the presence of basal-predominant hypertrophy, an adequate diameter of the septal artery (ranging from 1.25 mm to 2.5 mm), and the absence of concurrent surgical indications [110]. Notably, septal measurements of less than 2.5 mm are associated with a greater likelihood of benefiting from alcohol septal ablation. In contrast, cases exhibiting massive or diffuse septal hypertrophy extending into the mid-ventricle are better addressed through surgical resection, which effectively eliminates all hemodynamic gradients across the entire septal length [110].

Before initiating an ASA procedure, a transvenous pacemaker is typically positioned to mitigate the significant risk of conduction abnormalities, which ranges from 8% to 15%. The most prevalent conduction disorders observed in this context include right bundle branch block (RBBB), which occurs in approximately 50% to 70% of patients, and transient intraprocedural CHB resulting from tissue edema secondary to localized myocardial infarction [110]. Post-procedural improvements in gradients are not immediate, and the full benefits from ASA may take up to 1 year [110].

In centers with requisite expertise, ASA demonstrates a favorable procedural safety profile with perioperative mortality rates comparable to SM at approximately <1% [2]. However, longitudinal outcomes data reveal a significant divergence in survival trajectories, with SM conferring superior long-term survival at the 10-year follow-up interval (8.2% mortality) compared to ASA cohorts (26.1% mortality) [2, 111, 112]. Refer to Table 3 (Ref. [8, 104, 105, 110, 113, 114]) for the comparison between ASA and SM.

**Table 3.** **Comparison of surgical myectomy versus alcohol septal ablation**.

	Advantages	Disadvantages
Surgical myectomy [8, 104, 105, 110, 113, 114]	- More effective than ASA, with reduced residual outflow tract gradients	- Longer recovery time
	- Addresses mid-ventricular and apical hypertrophy	- More invasive
	- Preferred for massive hypertrophy (>30 mm)	- Expertise limited to few HCM centers of excellence
	- Does not form myocardial scar	- Left bundle branch more likely to be damaged, posing risk of complete heart block in patients with baseline right bundle branch block
	- Addresses additional problems (primary mitral disease, atrial fibrillation via Maze procedure, multivessel coronary artery disease, etc.)	- Less likely to result in damage to the coronary arteries
Alcohol septal ablation [8, 104, 105, 110, 113, 114]	- Shorter recovery time	- Less effective than SM
	- Less invasive	- Higher residual outflow tract gradients compared to SM
	- More available than SM	- Not preferred if hypertrophy extends to mid-ventricle
	- Preferred in elderly patients with minimal hypertrophy	- Requires adequate septal perforator artery length in the region of interest
	- Comparable perioperative mortality to SM at centers of excellence	- Increased risk of ventricular arrhythmias due to scar
		- Higher likelihood of developing complete heart block

ASA, alcohol septal ablation; HCM, hypertrophic cardiomyopathy; SM, surgical myectomy.

### 7.3 Emerging Techniques

In addition to SM and ASA, there are several emerging minimally invasive techniques for HCM management. Percutaneous intramyocardial septal radiofrequency ablation (PIMSRA) was evaluated in the largest cohort study involving 200 patients with drug-refractory symptomatic oHCM, and a significant overall reduction in the LVOT gradient was noted. However, 4% of patients continued to experience exertional chest pain or dyspnea despite the procedure. Additionally, 5.5% of patients developed permanent RBBB, while 1% experienced LBBB. Notably, no patients required permanent pacemaker implantation after the procedure. These findings suggest that PIMSRA may be an effective option for treating LVOT obstruction and providing symptomatic relief [115]. Additional procedures include transapical beating-heart septal myectomy, a minimally invasive technique for septal reduction that does not require cardiopulmonary bypass or a median sternotomy. Another option is the thoracoscopic Morrow procedure, which has also been demonstrated to provide significant symptomatic relief using a minimally invasive surgical approach [116, 117]. These emerging techniques offer favorable treatment options with minimized surgical trauma, and larger studies with long-term data will be needed to compare the efficacy of the minimally invasive techniques against the traditional SM and ASA procedures.

## 8. Nonobstructive Hypertrophic Cardiomyopathy

Nonobstructive HCM is a common subset representing patients without resting LVOT gradients or dynamic outflow tract obstruction. Nonobstructive HCM physiology is generally well-tolerated, and most patients are either asymptomatic or experience minimal symptoms, such as mild exertional dyspnea, chest discomfort, or fatigue. Symptoms can stem from diastolic dysfunction leading to increased left ventricular filling pressures, microvascular dysfunction, coronary artery disease, or heart failure.

However, the management of nonobstructive HCM remains challenging since there is currently a lack of clinical trials evaluating the long-term outcomes of medical management in this subset. BBs and CCBs are used as first-line agents in treating symptomatic nonobstructive HCM since they lower heart rate and improve LV filling with decreased LV diastolic pressures. Per the 2024 ACC/AHA HCM Guideline, an oral diuretic can also be considered for symptomatic relief in volume overload conditions (class of recommendation IIa) [2]. The benefits of the treatment of asymptomatic nonobstructive HCM are not well-studied and are not recommended presently.

A minor subset (5–10%) of nonobstructive patients can progress to advanced heart failure stages (NYHA classes III/IV) with symptoms refractory to pharmacological therapy and may require consideration for heart transplant [118].

## 9. Special Considerations

### 9.1 Advanced Heart Failure

In HCM patients with systolic dysfunction, defined as a LVEF of less than 50%, guideline-directed medical therapy (GDMT) should be initiated promptly according to the 2022 ACC/AHA Heart Failure Guideline. HCM patients with LVEF <35% are especially at a high risk of ventricular arrhythmias and death [2]. An ischemic evaluation should be performed in the setting of new systolic dysfunction to assess for concomitant etiologies such as obstructive coronary artery disease. Additionally, harmful ionotropic agents (verapamil, diltiazem, disopyramide) and cardiac myosin inhibitors should be discontinued in patients with LVEF <50%; ICD implantation for primary prevention of SCD should also be considered in this cohort. Most importantly, CPET should be performed to assess parameters of functional limitations such as peak oxygen consumption, minute ventilation to CO_2_ production, and ventilatory anaerobic threshold, and to refer the appropriate subset of patients for heart transplantation consideration [2]. As per the United Network for Organ Sharing data, the survival rates for HCM patients after heart transplant are 85%, 75%, and 61% at 1, 5, and 10 years, respectively [118]. Therefore, given these decent survival rates post-transplant, patients with severe or refractory HCM not meeting criteria for SRT with NYHA III/IV symptoms despite maximally tolerated GDMT should be considered for heart transplantation, with consideration of a left ventricular assist device as a bridge to transplantation [2].

### 9.2 Apical Hypertrophic Cardiomyopathy 

Previously thought to be a benign condition without a significant increase in mortality risk, recent data suggest that 1 in 3 patients with apical HCM (aHCM) may experience adverse life events such as malignant ventricular arrhythmias, SCD, and heart failure [119]. Recent data also reveals a mortality rate ranging from 0.5% to 4.8%, similar to that of typical HCM patients [119, 120]. In the absence of large randomized clinical trials, medical therapy in patients with aHCM is largely limited and primarily based on the management of classic HCM [120]. However, surgical options such as transapical myectomy and the novel transapical beating-heart septal myectomy may significantly benefit long-term survivability [120]. Lastly, while risk scores have been made to predict adverse events such as death, need for transplant, or ICD shocks, additional research is required to account for newer predictors of outcomes such as LGE [71].

### 9.3 Atrial Fibrillation

The risk of stroke is significantly higher in patients with HCM, and in cases of AF, anticoagulation is highly recommended regardless of the CHADS2-VASc score. Per the 2024 ACC/AHA HCM Guideline, direct-acting oral anticoagulants are the first-line option, and Vitamin K antagonists are the second-line treatment option. Regarding management, utilizing BBs, verapamil, or diltiazem as rate control agents is acceptable. In cases of poorly tolerated AF, a rhythm control strategy can be pursued with amiodarone, which is considered an effective agent in HCM patients. Alternatively, catheter ablation can be considered in patients with severely symptomatic AF, but HCM patients have twice the risk of relapse when compared to patients without HCM, likely due to a higher degree of electrophysiologic and structural remodeling noted in these patients [121].

### 9.4 Physical Activity

As per the 2024 ACC/AHA HCM Guideline, HCM patients should be counseled to engage in mild to moderate intensity recreational exercise to optimize their cardiovascular health and enhance their overall quality of life. The decision to participate in vigorous-intensity exercise, especially for athletes, relies on an individualized shared decision-making approach between the patient and an HCM expert, with extensive consideration of the potential risks and benefits. ICD implantation solely for participation in competitive sports is not recommended unless patients meet the clinical criteria described in Section 6.

### 9.5 Psychological Burden on Families

Families affected by HCM bear a significant psychological burden that affects not only the diagnosed individual but also their relatives. Research has shown that asymptomatic gene-positive relatives, known as silent gene carriers, face various psychological challenges. These include shock, worry, and uncertainty about their disease status, which can lead to anxiety and depression, ranging from minimal to severe levels [122]. Additionally, relatives of silent gene carriers often seek genetic testing primarily out of concern for their loved ones rather than personal benefit [122]. Moreover, the interpretation of positive gene results among silent carriers varies widely, with some patients viewing themselves as carriers without personal risk.

In contrast, others believe they have a serious heart condition, resulting in heightened anxiety and paranoia [122]. This unhealthy thought process leads to significant behavioral changes in some patients and influences career choices, physical activity, insurance decisions, and family planning [122]. Therefore, it is essential to provide adequate counseling and clearly communicate the meaning of genetic test results and their implications to patients to alleviate any psychological burden faced by families navigating this complex disease.

## 10. Future Perspectives

The current understanding of the pathophysiology of HCM, prominently at the molecular level, continues to evolve, and the genomic database continues to expand. It is crucial to elucidate further the complex genetics in HCM, which will lead to advances in the optimization of family screening and the development of appropriate risk stratification algorithms. There is increased interest in investigating genotype-targeted therapies for HCM, such as the MyPEAK-1 study, which is a phase 1b clinical trial investigating the safety, pharmacodynamics, and tolerability of TN-201 in patients with symptomatic HCM due to mutation in MYBPC3 [10, 123]. TN-201 is a recombinant adeno-associated virus serotype 9, which contains a myosin-binding protein c transgene, and the purpose of this investigational gene therapy is to assess if delivery of a functional MYBPC3 gene to the myocardium can aid in restoring normal heart function. The use of gene editing therapies in humans presents several challenges and limitations. These include the potential for off-target effects, which may increase the risk of cancer, the immunogenicity of the delivery vector, and the need to optimize delivery to cardiomyocytes. However, the outcomes of the MyPEAK-1 trial could open the door for future genome-targeting therapies [10].

Additionally, several studies have elucidated the role of prognostic biomarkers in HCM, most notably high sensitivity troponin T and NT-proBNP, with elevated levels reflecting increased myocardial wall stress and cardiac remodeling, which has been associated with adverse outcomes in patients with HCM. Extensive prospective studies are required to explore the role of biomarkers in disease progression and classify their impact in genotype-positive, phenotype-negative family members [124, 125]. Furthermore, integration of AI in imaging is also being utilized in HCM as mentioned in Section 3.2. The ongoing studies on AI-derived imaging analysis offer a promising approach to HCM diagnosis and monitoring of disease progression [97].

On the other hand, HCM molecular pathway targeting has led to the introduction of cardiac myosin inhibitors such as mavacamten and aficamten. The ODYSSEY-HCM (A Study of Mavacamten in Nonobstructive HCM) is a phase 3 randomized, double-blind, multicenter trial that investigated the efficacy of mavacamten in nonobstructive HCM patients on the following two primary end points: change from baseline to week 48 in Kansas City Cardiomyopathy Questionnaire-23 Clinical Summary Score (KCCQ-23 CSS) and peak oxygen consumption [97]. However, early updates from the phase 3 clinical trial have not shown promising results with mavacamten administration not leading to significant improvement in quality of life or peak oxygen consumption [126]. Additionally, ACACIA-HCM (Assessment Comparing Aficamten to Placebo on Cardiac Endpoints In Adults with Nonobstructive HCM) is a phase 3 multicenter clinical trial that will evaluate the efficacy of aficamten on improvement in health-related quality of life in patients with nonobstructive HCM [127].

Furthermore, a novel cardiac mitotrope agent, Ninerafaxstat, is being investigated in the IMPROVE-HCM phase 2 proof-of-concept clinical study to assess its efficacy in treating nonobstructive HCM [128]. Ninerafaxstat targets the energy metabolism pathway and improves the energy-depleted states in HCM patients; the agent was associated with enhanced ventilatory efficiency and exercise capacity in IMPROVE-HCM. Further clinical trials are warranted in the subsets of patients with nonobstructive HCM and heart failure with preserved ejection fraction to guide appropriate management in these patients to enhance their quality of life and functional performance.

## 11. Conclusion

HCM is a complex clinical entity with an intricate interplay between various genetic, environmental, physiologic, and lifestyle components. There is a broad spectrum of phenotypic manifestations, and appropriate screening and risk-stratification strategies should be employed in managing these patients. Treatment involves a multi-faceted approach including a combination of lifestyle, pharmacologic, and invasive surgical techniques, and the future seems promising with several clinical studies and novel agents on the horizon.

## References

[1] Braunwald E (2012). Hypertrophic cardiomyopathy: The first century 1869-1969. *Global Cardiology Science & Practice*.

[2] Ommen SR, Ho CY, Asif IM, Balaji S, Burke MA, Day SM (2024). 2024 AHA/ACC/AMSSM/HRS/PACES/SCMR Guideline for the Management of Hypertrophic Cardiomyopathy: A Report of the American Heart Association/American College of Cardiology Joint Committee on Clinical Practice Guidelines. *Circulation*.

[3] Das K J, Ingles J, Bagnall RD, Semsarian C (2014). Determining pathogenicity of genetic variants in hypertrophic cardiomyopathy: importance of periodic reassessment. *Genetics in Medicine*.

[4] Braunwald E (2024). Hypertrophic Cardiomyopathy: A Brief Overview. *The American Journal of Cardiology*.

[5] Maron BJ, Desai MY, Nishimura RA, Spirito P, Rakowski H, Towbin JA (2022). Diagnosis and Evaluation of Hypertrophic Cardiomyopathy: JACC State-of-the-Art Review. *Journal of the American College of Cardiology*.

[6] Marian AJ, Braunwald E (2017). Hypertrophic Cardiomyopathy: Genetics, Pathogenesis, Clinical Manifestations, Diagnosis, and Therapy. *Circulation Research*.

[7] Maron BJ, Ommen SR, Semsarian C, Spirito P, Olivotto I, Maron MS (2014). Hypertrophic cardiomyopathy: present and future, with translation into contemporary cardiovascular medicine. *Journal of the American College of Cardiology*.

[8] Maron BJ (2018). Clinical Course and Management of Hypertrophic Cardiomyopathy. *The New England Journal of Medicine*.

[9] Glavaški M, Velicki L, Vučinić N (2023). Hypertrophic Cardiomyopathy: Genetic Foundations, Outcomes, Interconnections, and Their Modifiers. *Medicina*.

[10] Lopes LR, Ho CY, Elliott PM (2024). Genetics of hypertrophic cardiomyopathy: established and emerging implications for clinical practice. *European Heart Journal*.

[11] Ingles J, Burns C, Bagnall RD, Lam L, Yeates L, Sarina T (2017). Nonfamilial Hypertrophic Cardiomyopathy: Prevalence, Natural History, and Clinical Implications. *Circulation. Cardiovascular Genetics*.

[12] Maron MS, Olivotto I, Zenovich AG, Link MS, Pandian NG, Kuvin JT (2006). Hypertrophic cardiomyopathy is predominantly a disease of left ventricular outflow tract obstruction. *Circulation*.

[13] Wigle ED, Rakowski H, Kimball BP, Williams WG (1995). Hypertrophic cardiomyopathy. Clinical spectrum and treatment. *Circulation*.

[14] Sherrid MV, Balaram S, Kim B, Axel L, Swistel DG (2016). The Mitral Valve in Obstructive Hypertrophic Cardiomyopathy: A Test in Context. *Journal of the American College of Cardiology*.

[15] Groarke JD, Galazka PZ, Cirino AL, Lakdawala NK, Thune JJ, Bundgaard H (2018). Intrinsic mitral valve alterations in hypertrophic cardiomyopathy sarcomere mutation carriers. *European Heart Journal. Cardiovascular Imaging*.

[16] Captur G, Ho CY, Schlossarek S, Kerwin J, Mirabel M, Wilson R (2016). The embryological basis of subclinical hypertrophic cardiomyopathy. *Scientific Reports*.

[17] Velicki L, Jakovljevic DG, Preveden A, Golubovic M, Bjelobrk M, Ilic A (2020). Genetic determinants of clinical phenotype in hypertrophic cardiomyopathy. *BMC Cardiovascular Disorders*.

[18] Hong JH, Schaff HV, Nishimura RA, Abel MD, Dearani JA, Li Z (2016). Mitral Regurgitation in Patients With Hypertrophic Obstructive Cardiomyopathy: Implications for Concomitant Valve Procedures. *Journal of the American College of Cardiology*.

[19] Yu EH, Omran AS, Wigle ED, Williams WG, Siu SC, Rakowski H (2000). Mitral regurgitation in hypertrophic obstructive cardiomyopathy: relationship to obstruction and relief with myectomy. *Journal of the American College of Cardiology*.

[20] Nagueh SF, Phelan D, Abraham T, Armour A, Desai MY, Dragulescu A (2022). Recommendations for Multimodality Cardiovascular Imaging of Patients with Hypertrophic Cardiomyopathy: An Update from the American Society of Echocardiography, in Collaboration with the American Society of Nuclear Cardiology, the Society for Cardiovascular Magnetic Resonance, and the Society of Cardiovascular Computed Tomography. *Journal of the American Society of Echocardiography*.

[21] Hang D, Schaff HV, Nishimura RA, Lahr BD, Abel MD, Dearani JA (2019). Accuracy of Jet Direction on Doppler Echocardiography in Identifying the Etiology of Mitral Regurgitation in Obstructive Hypertrophic Cardiomyopathy. *Journal of the American Society of Echocardiography*.

[22] Wu H, Yang H, Rhee JW, Zhang JZ, Lam CK, Sallam K (2019). Modelling diastolic dysfunction in induced pluripotent stem cell-derived cardiomyocytes from hypertrophic cardiomyopathy patients. *European Heart Journal*.

[23] Maron MS, Olivotto I, Maron BJ, Prasad SK, Cecchi F, Udelson JE (2009). The case for myocardial ischemia in hypertrophic cardiomyopathy. *Journal of the American College of Cardiology*.

[24] Pelliccia F, Cecchi F, Olivotto I, Camici PG (2022). Microvascular Dysfunction in Hypertrophic Cardiomyopathy. *Journal of Clinical Medicine*.

[25] Malahfji M, Senapati A, Debs D, Angulo C, Zhan Y, Nagueh SF (2020). Examining the impact of inducible ischemia on myocardial fibrosis and exercise capacity in hypertrophic cardiomyopathy. *Scientific Reports*.

[26] Coleman JA, Ashkir Z, Raman B, Bueno-Orovio A (2023). Mechanisms and prognostic impact of myocardial ischaemia in hypertrophic cardiomyopathy. *The International Journal of Cardiovascular Imaging*.

[27] Weissler-Snir A, Saberi S, Wong TC, Pantazis A, Owens A, Leunig A (2024). Atrial Fibrillation in Hypertrophic Cardiomyopathy. *JACC. Advances*.

[28] Coppini R, Santini L, Olivotto I, Ackerman MJ, Cerbai E (2020). Abnormalities in sodium current and calcium homoeostasis as drivers of arrhythmogenesis in hypertrophic cardiomyopathy. *Cardiovascular Research*.

[29] Santini L, Coppini R, Cerbai E (2021). Ion Channel Impairment and Myofilament Ca2+ Sensitization: Two Parallel Mechanisms Underlying Arrhythmogenesis in Hypertrophic Cardiomyopathy. *Cells*.

[30] Wolf CM, Moskowitz IPG, Arno S, Branco DM, Semsarian C, Bernstein SA (2005). Somatic events modify hypertrophic cardiomyopathy pathology and link hypertrophy to arrhythmia. *Proceedings of the National Academy of Sciences of the United States of America*.

[31] Santoro F, Mango F, Mallardi A, D’Alessandro D, Casavecchia G, Gravina M (2023). Arrhythmic Risk Stratification among Patients with Hypertrophic Cardiomyopathy. *Journal of Clinical Medicine*.

[32] Nollet EE, Duursma I, Rozenbaum A, Eggelbusch M, Wüst RCI, Schoonvelde SAC (2023). Mitochondrial dysfunction in human hypertrophic cardiomyopathy is linked to cardiomyocyte architecture disruption and corrected by improving NADH-driven mitochondrial respiration. *European Heart Journal*.

[33] Ranjbarvaziri S, Kooiker KB, Ellenberger M, Fajardo G, Zhao M, Vander Roest AS (2021). Altered Cardiac Energetics and Mitochondrial Dysfunction in Hypertrophic Cardiomyopathy. *Circulation*.

[34] Dass S, Cochlin LE, Suttie JJ, Holloway CJ, Rider OJ, Carden L (2015). Exacerbation of cardiac energetic impairment during exercise in hypertrophic cardiomyopathy: a potential mechanism for diastolic dysfunction. *European Heart Journal*.

[35] Ommen SR, Nishimura RA, Schaff HV, Dearani JA (2025). Hypertrophic Cardiomyopathy: State of the Art. *Mayo Clinic Proceedings*.

[36] Young L, Smedira NG, Tower-Rader A, Lever H, Desai MY (2018). Hypertrophic cardiomyopathy: A complex disease. *Cleveland Clinic Journal of Medicine*.

[37] Finocchiaro G, Sheikh N, Biagini E, Papadakis M, Maurizi N, Sinagra G (2020). The electrocardiogram in the diagnosis and management of patients with hypertrophic cardiomyopathy. *Heart Rhythm*.

[38] Ko WY, Siontis KC, Attia ZI, Carter RE, Kapa S, Ommen SR (2020). Detection of Hypertrophic Cardiomyopathy Using a Convolutional Neural Network-Enabled Electrocardiogram. *Journal of the American College of Cardiology*.

[39] Maron BJ, Maron MS, Semsarian C (2012). Genetics of hypertrophic cardiomyopathy after 20 years: clinical perspectives. *Journal of the American College of Cardiology*.

[40] Siontis KC, Liu K, Bos JM, Attia ZI, Cohen-Shelly M, Arruda-Olson AM (2021). Detection of hypertrophic cardiomyopathy by an artificial intelligence electrocardiogram in children and adolescents. *International Journal of Cardiology*.

[41] Carrick RT, Ahamed H, Sung E, Maron MS, Madias C, Avula V (2024). Identification of high-risk imaging features in hypertrophic cardiomyopathy using electrocardiography: A deep-learning approach. *Heart Rhythm*.

[42] Robyns T, Breckpot J, Nuyens D, Vandenberk B, Corveleyn A, Kuiperi C (2020). Clinical and ECG variables to predict the outcome of genetic testing in hypertrophic cardiomyopathy. *European Journal of Medical Genetics*.

[43] Abraham MR, Abraham TP (2024). Role of Imaging in the Diagnosis, Evaluation, and Management of Hypertrophic Cardiomyopathy. *The American Journal of Cardiology*.

[44] Bos JM, Towbin JA, Ackerman MJ (2009). Diagnostic, prognostic, and therapeutic implications of genetic testing for hypertrophic cardiomyopathy. *Journal of the American College of Cardiology*.

[45] Leggit JC, Whitaker D (2022). Diagnosis and Management of Hypertrophic Cardiomyopathy: Updated Guidelines From the ACC/AHA. *American Family Physician*.

[46] Quarta G, Aquaro GD, Pedrotti P, Pontone G, Dellegrottaglie S, Iacovoni A (2018). Cardiovascular magnetic resonance imaging in hypertrophic cardiomyopathy: the importance of clinical context. *European Heart Journal. Cardiovascular Imaging*.

[47] Rowin EJ, Maron MS (2016). The Role of Cardiac MRI in the Diagnosis and Risk Stratification of Hypertrophic Cardiomyopathy. *Arrhythmia & Electrophysiology Review*.

[48] Hindieh W, Weissler-Snir A, Hammer H, Adler A, Rakowski H, Chan RH (2017). Discrepant Measurements of Maximal Left Ventricular Wall Thickness Between Cardiac Magnetic Resonance Imaging and Echocardiography in Patients With Hypertrophic Cardiomyopathy. *Circulation. Cardiovascular Imaging*.

[49] Webb J, Villa A, Bekri I, Shome J, Teall T, Claridge S (2017). Usefulness of Cardiac Magnetic Resonance Imaging to Measure Left Ventricular Wall Thickness for Determining Risk Scores for Sudden Cardiac Death in Patients With Hypertrophic Cardiomyopathy. *The American Journal of Cardiology*.

[50] Weng Z, Yao J, Chan RH, He J, Yang X, Zhou Y (2016). Prognostic Value of LGE-CMR in HCM: A Meta-Analysis. *JACC. Cardiovascular Imaging*.

[51] Sorajja P, Borlaug BA, Dimas VV, Fang JC, Forfia PR, Givertz MM (2017). SCAI/HFSA clinical expert consensus document on the use of invasive hemodynamics for the diagnosis and management of cardiovascular disease. *Catheterization and Cardiovascular Interventions*.

[52] Coats CJ, Rantell K, Bartnik A, Patel A, Mist B, McKenna WJ (2015). Cardiopulmonary Exercise Testing and Prognosis in Hypertrophic Cardiomyopathy. *Circulation. Heart Failure*.

[53] Ciampi Q, Olivotto I, Peteiro J, D’Alfonso MG, Mori F, Tassetti L (2021). Prognostic Value of Reduced Heart Rate Reserve during Exercise in Hypertrophic Cardiomyopathy. *Journal of Clinical Medicine*.

[54] Rodrigues T, Raposo SC, Brito D, Lopes LR (2021). Prognostic relevance of exercise testing in hypertrophic cardiomyopathy. A systematic review. *International Journal of Cardiology*.

[55] Bayonas-Ruiz A, Muñoz-Franco FM, Ferrer V, Pérez-Caballero C, Sabater-Molina M, Tomé-Esteban MT (2021). Cardiopulmonary Exercise Test in Patients with Hypertrophic Cardiomyopathy: A Systematic Review and Meta-Analysis. *Journal of Clinical Medicine*.

[56] Finocchiaro G, Haddad F, Knowles JW, Caleshu C, Pavlovic A, Homburger J (2015). Cardiopulmonary responses and prognosis in hypertrophic cardiomyopathy: a potential role for comprehensive noninvasive hemodynamic assessment. *JACC. Heart Failure*.

[57] Ishibashi-Ueda H, Matsuyama TA, Ohta-Ogo K, Ikeda Y (2017). Significance and Value of Endomyocardial Biopsy Based on Our Own Experience. *Circulation Journal*.

[58] Ehsan M, Jiang H, L Thomson K, Gehmlich K (2017). When signalling goes wrong: pathogenic variants in structural and signalling proteins causing cardiomyopathies. *Journal of Muscle Research and Cell Motility*.

[59] Shirani J, Pick R, Roberts WC, Maron BJ (2000). Morphology and significance of the left ventricular collagen network in young patients with hypertrophic cardiomyopathy and sudden cardiac death. *Journal of the American College of Cardiology*.

[60] Fontana M, Chung R, Hawkins PN, Moon JC (2015). Cardiovascular magnetic resonance for amyloidosis. *Heart Failure Reviews*.

[61] Flodrova P, Flodr P, Pika T, Vymetal J, Holub D, Dzubak P (2018). Cardiac amyloidosis: from clinical suspicion to morphological diagnosis. *Pathology*.

[62] McLendon PM, Robbins J (2011). Desmin-related cardiomyopathy: an unfolding story. *American Journal of Physiology. Heart and Circulatory Physiology*.

[63] Wechalekar AD, Gillmore JD, Hawkins PN (2016). Systemic amyloidosis. *Lancet*.

[64] Kubánek M, Schimerová T, Piherová L, Brodehl A, Krebsová A, Ratnavadivel S (2020). Desminopathy: Novel Desmin Variants, a New Cardiac Phenotype, and Further Evidence for Secondary Mitochondrial Dysfunction. *Journal of Clinical Medicine*.

[65] Iorio A, Lucà F, Pozzi A, Rao CM, Chimenti C, Di Fusco SA (2024). Anderson-Fabry Disease: Red Flags for Early Diagnosis of Cardiac Involvement. *Diagnostics*.

[66] Wilde AAM, Semsarian C, Márquez MF, Sepehri Shamloo A, Ackerman MJ, Ashley EA (2022). European Heart Rhythm Association (EHRA)/Heart Rhythm Society (HRS)/Asia Pacific Heart Rhythm Society (APHRS)/Latin American Heart Rhythm Society (LAHRS) Expert Consensus Statement on the State of Genetic Testing for Cardiac Diseases. *Heart Rhythm*.

[67] Finocchiaro G, Westaby J, Sheppard MN, Papadakis M, Sharma S (2024). Sudden Cardiac Death in Young Athletes: JACC State-of-the-Art Review. *Journal of the American College of Cardiology*.

[68] Peterson DF, Kucera K, Thomas LC, Maleszewski J, Siebert D, Lopez-Anderson M (2021). Aetiology and incidence of sudden cardiac arrest and death in young competitive athletes in the USA: a 4-year prospective study. *British Journal of Sports Medicine*.

[69] Maron BJ, Rowin EJ, Maron MS (2019). Paradigm of Sudden Death Prevention in Hypertrophic Cardiomyopathy. *Circulation Research*.

[70] Nistri S, Olivotto I, Betocchi S, Losi MA, Valsecchi G, Pinamonti B (2006). Prognostic significance of left atrial size in patients with hypertrophic cardiomyopathy (from the Italian Registry for Hypertrophic Cardiomyopathy). *The American Journal of Cardiology*.

[71] Hajj-Ali A, Gaballa A, Akintoye E, Jadam S, Ramchand J, Xu B (2024). Long-Term Outcomes of Patients With Apical Hypertrophic Cardiomyopathy Utilizing a New Risk Score. *JACC. Advances*.

[72] Chan RH, van der Wal L, Liberato G, Rowin E, Soslow J, Maskatia S (2024). Myocardial Scarring and Sudden Cardiac Death in Young Patients With Hypertrophic Cardiomyopathy: A Multicenter Cohort Study. *JAMA Cardiology*.

[73] Chiotis S, Doundoulakis I, Zgouridou A, Piperis C, Raptis D, Peletidi A (2025). Predictors of Arrhythmic Events in Hypertrophic Cardiomyopathy Patients with an Implantable Cardioverter-Defibrillator: A Systematic Review and Meta-Analysis. *European Heart Journal. Quality of Care & Clinical Outcomes*.

[74] O’Mahony C, Jichi F, Pavlou M, Monserrat L, Anastasakis A, Rapezzi C (2014). A novel clinical risk prediction model for sudden cardiac death in hypertrophic cardiomyopathy (HCM risk-SCD). *European Heart Journal*.

[75] Maron MS, Rowin EJ, Wessler BS, Mooney PJ, Fatima A, Patel P (2019). Enhanced American College of Cardiology/American Heart Association Strategy for Prevention of Sudden Cardiac Death in High-Risk Patients With Hypertrophic Cardiomyopathy. *JAMA Cardiology*.

[76] Sylvester J, Seidenberg P, Silvis M (2014). The dilemma of genotype positive-phenotype negative hypertrophic cardiomyopathy. *Current Sports Medicine Reports*.

[77] Christensen EB, Vissing CR, Silajdzija E, Mills HL, Thune JJ, Larroudé C (2025). Long-term incidence of implantable cardioverter-defibrillator therapy in patients with hypertrophic cardiomyopathy: analysis of appropriate and inappropriate interventions. *Heart*.

[78] Francia P, Olivotto I, Lambiase PD, Autore C (2022). Implantable cardioverter-defibrillators for hypertrophic cardiomyopathy: The Times They Are a-Changin’. *Europace*.

[79] Knops RE, Olde Nordkamp LRA, Delnoy PPHM, Boersma LVA, Kuschyk J, El-Chami MF (2020). Subcutaneous or Transvenous Defibrillator Therapy. *The New England Journal of Medicine*.

[80] Liebregts M, Vriesendorp PA, Mahmoodi BK, Schinkel AFL, Michels M, ten Berg JM (2015). A Systematic Review and Meta-Analysis of Long-Term Outcomes After Septal Reduction Therapy in Patients With Hypertrophic Cardiomyopathy. *JACC. Heart Failure*.

[81] Maron BJ, Spirito P, Ackerman MJ, Casey SA, Semsarian C, Estes NAM (2013). Prevention of sudden cardiac death with implantable cardioverter-defibrillators in children and adolescents with hypertrophic cardiomyopathy. *Journal of the American College of Cardiology*.

[82] Zhu M, Reyes KRL, Bilgili G, Siegel RJ, Lee Claggett B, Wong TC (2023). Medical Therapies to Improve Left Ventricular Outflow Obstruction and Diastolic Function in Hypertrophic Cardiomyopathy. *JACC. Advances*.

[83] Coppini R, Ferrantini C, Pioner JM, Santini L, Wang ZJ, Palandri C (2019). Electrophysiological and Contractile Effects of Disopyramide in Patients With Obstructive Hypertrophic Cardiomyopathy: A Translational Study. *JACC. Basic to Translational Science*.

[84] Massera D, Sherrid MV, Adlestein E, Bokhari N, Alvarez IC, Wu WY (2025). Disopyramide Revisited for Treatment of Symptomatic Obstructive Hypertrophic Cardiomyopathy: Efficacy and Safety in Patients Treated for at Least 5 Years. *Journal of the American Heart Association*.

[85] Braunwald E, Saberi S, Abraham TP, Elliott PM, Olivotto I (2023). Mavacamten: a first-in-class myosin inhibitor for obstructive hypertrophic cardiomyopathy. *European Heart Journal*.

[86] Olivotto I, Oreziak A, Barriales-Villa R, Abraham TP, Masri A, Garcia-Pavia P (2020). Mavacamten for treatment of symptomatic obstructive hypertrophic cardiomyopathy (EXPLORER-HCM): a randomised, double-blind, placebo-controlled, phase 3 trial. *Lancet*.

[87] Garcia-Pavia P, Oręziak A, Masri A, Barriales-Villa R, Abraham TP, Owens AT (2024). Long-term effect of mavacamten in obstructive hypertrophic cardiomyopathy. *European Heart Journal*.

[88] Maron MS, Masri A, Nassif ME, Barriales-Villa R, Arad M, Cardim N (2024). Aficamten for Symptomatic Obstructive Hypertrophic Cardiomyopathy. *The New England Journal of Medicine*.

[89] Maron MS, Masri A, Nassif ME, Barriales-Villa R, Abraham TP, Arad M (2024). Impact of Aficamten on Disease and Symptom Burden in Obstructive Hypertrophic Cardiomyopathy: Results From SEQUOIA-HCM. *Journal of the American College of Cardiology*.

[90] Masri A, Cardoso RN, Abraham TP, Claggett BL, Coats CJ, Hegde SM (2024). Effect of Aficamten on Cardiac Structure and Function in Obstructive Hypertrophic Cardiomyopathy: SEQUOIA-HCM CMR Substudy. *Journal of the American College of Cardiology*.

[91] Kawas RF, Anderson RL, Ingle SRB, Song Y, Sran AS, Rodriguez HM (2017). A small-molecule modulator of cardiac myosin acts on multiple stages of the myosin chemomechanical cycle. *The Journal of Biological Chemistry*.

[92] Desai MY, Owens A, Wolski K, Geske JB, Saberi S, Wang A (2023). Mavacamten in Patients With Hypertrophic Cardiomyopathy Referred for Septal Reduction: Week 56 Results From the VALOR-HCM Randomized Clinical Trial. *JAMA Cardiology*.

[93] Desai MY, Owens A, Geske JB, Wolski K, Naidu SS, Smedira NG (2022). Myosin Inhibition in Patients With Obstructive Hypertrophic Cardiomyopathy Referred for Septal Reduction Therapy. *Journal of the American College of Cardiology*.

[94] Desai MY, Owens A, Saberi S, Wang A, Wolski K, Cremer PC (2025). Long-Term Effects of Mavacamten on Patients Based on Hypertrophic Cardiomyopathy Pathogenic Genetic Variant Status: Insights From VALOR-HCM Trial. *Circulation. Genomic and Precision Medicine*.

[95] Tian Z, Li L, Li X, Wang J, Zhang Q, Li Z (2023). Effect of Mavacamten on Chinese Patients With Symptomatic Obstructive Hypertrophic Cardiomyopathy: The EXPLORER-CN Randomized Clinical Trial. *JAMA Cardiology*.

[96] Bristol Myers Squibb (2025). Bristol Myers Squibb Provides Update on Phase 3 ODYSSEY-HCM Trial. https://news.bms.com/news/details/2025/Bristol-Myers-Squibb-Provides-Update-on-Phase-3-ODYSSEY-HCM-Trial/.

[97] Desai MY, Nissen SE, Abraham T, Olivotto I, Garcia-Pavia P, Lopes RD (2025). Mavacamten in Symptomatic Nonobstructive Hypertrophic Cardiomyopathy: Design, Rationale, and Baseline Characteristics of ODYSSEY-HCM. *JACC. Heart Failure*.

[98] Desai MY, Nissen SE, Abraham T, Owens A, Olivotto I, Executive Committee of the ODYSSEY-HCM trial (2025). Reply: Hypertrophic Cardiomyopathy With Midventricular Obstruction Is Not Nonobstructive Hypertrophic Cardiomyopathy. *JACC. Heart Failure*.

[99] FDA, CDER (2022). HIGHLIGHTS OF PRESCRIBING INFORMATION. https://www.accessdata.fda.gov/drugsatfda_docs/label/2022/214998s000lbl.pdf.

[100] Sherrod CF, Saberi S, Nassif ME, Claggett BL, Coats CJ, Garcia-Pavia P (2024). Effect of Aficamten on Health Status Outcomes in Obstructive Hypertrophic Cardiomyopathy: Results From SEQUOIA-HCM. *Journal of the American College of Cardiology*.

[101] Maron BJ, Dearani JA, Smedira NG, Schaff HV, Wang S, Rastegar H (2022). Ventricular Septal Myectomy for Obstructive Hypertrophic Cardiomyopathy (Analysis Spanning 60 Years Of Practice): AJC Expert Panel. *The American Journal of Cardiology*.

[102] MORROW AG, BROCKENBROUGH EC (1961). Surgical treatment of idiopathic hypertrophic subaortic stenosis: technic and hemodynamic results of subaortic ventriculomyotomy. *Annals of Surgery*.

[103] Holst KA, Hanson KT, Ommen SR, Nishimura RA, Habermann EB, Schaff HV (2019). Septal Myectomy in Hypertrophic Cardiomyopathy: National Outcomes of Concomitant Mitral Surgery. *Mayo Clinic Proceedings*.

[104] Nishimura RA, Seggewiss H, Schaff HV (2017). Hypertrophic Obstructive Cardiomyopathy: Surgical Myectomy and Septal Ablation. *Circulation Research*.

[105] Cui H, Schaff HV, Nishimura RA, Geske JB, Dearani JA, Lahr BD (2019). Conduction Abnormalities and Long-Term Mortality Following Septal Myectomy in Patients With Obstructive Hypertrophic Cardiomyopathy. *Journal of the American College of Cardiology*.

[106] Inestroza K, Mijares-Rojas I, Matute-Martínez C, Ergui I, Albosta M, Vergara-Sanchez C (2024). In-hospital outcomes of septal myectomy vs. alcohol septal ablation for hypertrophic cardiomyopathy with outflow tract obstruction: An update and insights from the national inpatient sample from 2011 to 2019. *Journal of Investigative Medicine*.

[107] Kotkar KD, Said SM, Dearani JA, Schaff HV (2017). Hypertrophic obstructive cardiomyopathy: the Mayo Clinic experience. *Annals of Cardiothoracic Surgery*.

[108] Rastegar H, Boll G, Rowin EJ, Dolan N, Carroll C, Udelson JE (2017). Results of surgical septal myectomy for obstructive hypertrophic cardiomyopathy: the Tufts experience. *Annals of Cardiothoracic Surgery*.

[109] Altibi AM, Ghanem F, Zhao Y, Elman M, Cigarroa J, Nazer B (2023). Hospital Procedural Volume and Clinical Outcomes Following Septal Reduction Therapy in Obstructive Hypertrophic Cardiomyopathy. *Journal of the American Heart Association*.

[110] Bali AD, Malik A, Naidu SS (2024). Treatment Strategies for Hypertrophic Cardiomyopathy: Alcohol Septal Ablation and Procedural Step-by-Step Technique. *The American Journal of Cardiology*.

[111] Nguyen A, Schaff HV, Hang D, Nishimura RA, Geske JB, Dearani JA (2019). Surgical myectomy versus alcohol septal ablation for obstructive hypertrophic cardiomyopathy: A propensity score-matched cohort. *The Journal of Thoracic and Cardiovascular Surgery*.

[112] Cui H, Schaff HV, Wang S, Lahr BD, Rowin EJ, Rastegar H (2022). Survival Following Alcohol Septal Ablation or Septal Myectomy for Patients With Obstructive Hypertrophic Cardiomyopathy. *Journal of the American College of Cardiology*.

[113] Valeti US, Nishimura RA, Holmes DR, Araoz PA, Glockner JF, Breen JF (2007). Comparison of surgical septal myectomy and alcohol septal ablation with cardiac magnetic resonance imaging in patients with hypertrophic obstructive cardiomyopathy. *Journal of the American College of Cardiology*.

[114] Alam M, Dokainish H, Lakkis N (2006). Alcohol septal ablation for hypertrophic obstructive cardiomyopathy: a systematic review of published studies. *Journal of Interventional Cardiology*.

[115] Zhou M, Ta S, Hahn RT, Hsi DH, Leon MB, Hu R (2022). Percutaneous Intramyocardial Septal Radiofrequency Ablation in Patients With Drug-Refractory Hypertrophic Obstructive Cardiomyopathy. *JAMA Cardiology*.

[116] Li J, Wei X (2024). Transapical beating-heart septal myectomy for hypertrophic cardiomyopathy with latent obstruction. *European Journal of Cardio-Thoracic Surgery*.

[117] Dong Z, Wang S, Liu Z, Han E, Wu C, Luo C (2024). An innovative minimally invasive approach for hypertrophic obstructive cardiomyopathy: Transaortic septal myectomy via right infra-axillary incision. *JTCVS Techniques*.

[118] Maron BJ, Desai MY, Nishimura RA, Spirito P, Rakowski H, Towbin JA (2022). Management of Hypertrophic Cardiomyopathy: JACC State-of-the-Art Review. *Journal of the American College of Cardiology*.

[119] Yin Y, Hu W, Zhang L, Wu D, Yang C, Ye X (2022). Clinical, echocardiographic and cardiac MRI predictors of outcomes in patients with apical hypertrophic cardiomyopathy. *The International Journal of Cardiovascular Imaging*.

[120] Li J, Fang J, Liu Y, Wei X (2024). Apical hypertrophic cardiomyopathy: pathophysiology, diagnosis and management. *Clinical Research in Cardiology*.

[121] Providencia R, Elliott P, Patel K, McCready J, Babu G, Srinivasan N (2016). Catheter ablation for atrial fibrillation in hypertrophic cardiomyopathy: a systematic review and meta-analysis. *Heart*.

[122] Bonner C, Spinks C, Semsarian C, Barratt A, Ingles J, McCaffery K (2018). Psychosocial Impact of a Positive Gene Result for Asymptomatic Relatives at Risk of Hypertrophic Cardiomyopathy. *Journal of Genetic Counseling*.

[123] (2024). MyPeak-1 Clinical Trial Treating HCM in Adults - Tenaya Therapeutics. https://hcmstudies.com/our-studies/mypeak-1/.

[124] Tahir UA, Kolm P, Kwong RY, Desai MY, Dolman SF, Deng S (2024). Protein Biomarkers of Adverse Clinical Features and Events in Sarcomeric Hypertrophic Cardiomyopathy. *Circulation. Heart Failure*.

[125] Jansen M, Algül S, Bosman LP, Michels M, van der Velden J, de Boer RA (2022). Blood-based biomarkers for the prediction of hypertrophic cardiomyopathy prognosis: a systematic review and meta-analysis. *ESC Heart Failure*.

[126] McKeown LA (2025). Mavacamten Strikes Out in Phase III Trial of Nonobstructive HCM. https://www.tctmd.com/news/mavacamten-strikes-out-phase-iii-trial-nonobstructive-hcm.

[127] (2022). Hypertrophic Cardiomyopathy Clinical Trials - Aficamten. https://cytokinetics.com/medicines-research/hypertrophic-cardiomyopathy-clinical-trials/.

[128] Maron MS, Mahmod M, Abd Samat AH, Choudhury L, Massera D, Phelan DMJ (2024). Safety and Efficacy of Metabolic Modulation With Ninerafaxstat in Patients With Nonobstructive Hypertrophic Cardiomyopathy. *Journal of the American College of Cardiology*.

